# Genesis of ultra-high-Ni olivine in high-Mg andesite lava triggered by seamount subduction

**DOI:** 10.1038/s41598-017-10276-3

**Published:** 2017-09-14

**Authors:** Tatsuji Nishizawa, Hitomi Nakamura, Tatiana Churikova, Boris Gordeychik, Osamu Ishizuka, Satoru Haraguchi, Takashi Miyazaki, Bogdan Stefanov Vaglarov, Qing Chang, Morihisa Hamada, Jun-Ichi Kimura, Kenta Ueki, Chiaki Toyama, Atsushi Nakao, Hikaru Iwamori

**Affiliations:** 1 Department of Earth and Planetary Sciences, Tokyo Institute of Technology, 2-12-1 Ookayama, Meguro-ku Tokyo, 152-8551 Japan; 2 Department of Solid Earth Geochemistry, Japan Agency for Marine-Earth Science and Technology, 2-15 Natsushima-cho, Yokosuka, Kanagawa 237-0061 Japan; 3 Chiba Institute of Technology, ORCeNG, 2-17-1 Tsudanuma, Narashino Chiba, 275-0016 Japan; 4 Institute of Volcanology and Seismology, Far East Branch, Russian Academy of Sciences, 9 Piip Boulevard, Petropavlovsk-Kamchatsky, 683006 Russia; 5 Institute of Experimental Mineralogy, Russian Academy of Sciences, 4 Academica Osypyana ul., Chernogolovka, Moscow region 142432 Russia; 6 Institute of Earthquake and Volcano Geology, Geological Survey of Japan, AIST, Central 7, 1-1-1 Higashi, Tsukuba Ibaraki, 305-8567 Japan; 7 R & D Center for Ocean Drilling Science, Japan Agency for Marine-Earth Science and Technology, 2-15 Natsushima-cho, Yokosuka, Kanagawa 237-0061 Japan; 8 Earthquake Research Institute, The University of Tokyo, 1-1-1 Yayoi, Bunkyo-ku Tokyo, 113-0032 Japan; 9 Institute of Geology and Geoinformation, Geological Survey of Japan, AIST, Central 7, 1-1-1 Higashi, Tsukuba, Ibaraki 305-8567 Japan

## Abstract

The Kamchatka Peninsula is a prominent and wide volcanic arc located near the northern edge of the Pacific Plate. It has highly active volcanic chains and groups, and characteristic lavas that include adakitic rocks. In the north of the peninsula adjacent to the triple junction, some additional processes such as hot asthenospheric injection around the slab edge and seamount subduction operate, which might enhance local magmatism. In the forearc area of the northeastern part of the peninsula, monogenetic volcanic cones dated at <1 Ma were found. Despite their limited spatiotemporal occurrence, remarkable variations were observed, including primitive basalt and high-Mg andesite containing high-Ni (up to 6300 ppm) olivine. The melting and crystallization conditions of these lavas indicate a locally warm slab, facilitating dehydration beneath the forearc region, and a relatively cold overlying mantle wedge fluxed heterogeneously by slab-derived fluids. It is suggested that the collapse of a subducted seamount triggered the ascent of Si-rich fluids to vein the wedge peridotite and formed a peridotite–pyroxenite source, causing the temporal evolution of local magmatism with wide compositional range.

## Introduction

**Figure 1:**
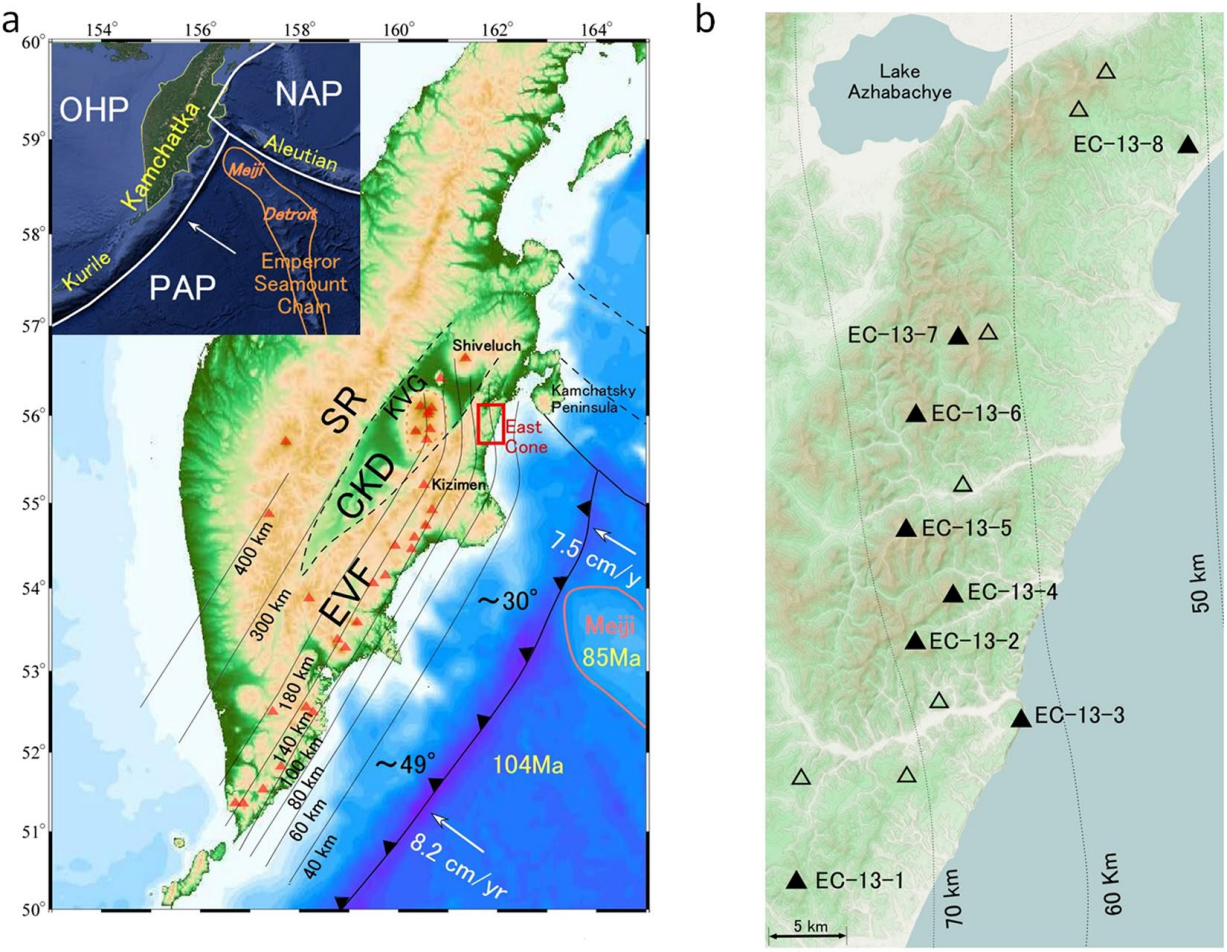
Plate tectonic setting and major geological structures of the Kamchatka Peninsula and detailed map of the studied area (EC). (**a**) The inset shows the plate tectonic setting and location of the Emperor Seamount Chain that connects to the Hawaiian hotspot. The North American Plate (NAP), Pacific Plate (PAP), and Okhotsk Plate (OHP) form a triple junction in this region. Plate configuration is after ref. 73. White arrows indicate the motion vector of the Pacific Plate. Red triangles indicate locations of active volcanoes. Thin lines with numbers show depth of subducted Pacific Plate^2^. Black dashed lines are from ref. 74. (**b**) Topographic map of studied area (EC). Black triangles indicate monogenetic cones of the EC, the true heights of which are 200–600 m and the relative elevation is ~200 m. The slab depth is 50–80 km (dotted lines)^2^ and the thickness of the crust is 25–30 km^75, 76^. Sampling was performed at the top and/or hillside on 8 cones (filled black triangles) from which 16 fresh lavas were obtained for use in this study. Figure 1a was generated by using Generic Mapping Tools^77^. The inset was captured from Google Earth™ with the map data provided by NOAA, U.S. Navy, NGA, GEBCO. Figure 1b was captured from OpenStreetMap with the map data provided by © OpenStreetMap contributors, CC-BY-SA. All figures were overlaid by using Canvas 11.

The Kamchatka Peninsula is one of the largest volcanic arcs in the world. It corresponds to the subduction of the northernmost part of the Pacific Plate and it comprises three volcanic chains^1^. The volcanic front is continuous from the Kurile Arc to the Eastern Volcanic Front (EVF, Fig. 1a). The volcanic front is bent toward the Kliuchevskoy Volcanic Group (KVG) at around 55°N along the 100–180-km slab-depth contour (Fig. 1a)^2^. The northern end of the volcanic front is the Shiveluch Volcano, which is located on the slab edge of the Pacific Plate where the mantle wedge opens to the north^3, 4^ and where asthenospheric flow around the slab edge might heat the slab and cause melting^3^. The extension of the Emperor Seamount Chain is subducted from the southeast^5, 6^, which contributes to the northward shallowing of the subduction dip angle^2^ and influences the magmatism in northern Kamchatka^6–8^. The Kamchatka Peninsula is a unique place that has undergone these extremely dynamic processes, where the geological structure and igneous materials continue to reflect the interaction between the subducting Pacific Plate and the overlying arc system via material cycling and structural evolution.

This study investigated the East Cone volcanic group^9, 10^ (hereafter, EC) in the northeastern forearc area in the northern termination of the EVF^1^ and to the east of the KVG. The EC is located ~60 km above the subducting slab, which is presumed old and cold (~100 Ma^11^). In this case, a supply of slab-derived fluid and corresponding mantle melting would be expected to form an arc volcanic zone in the region above where the depth of the slab is ≳100 km^12^, but not where the forearc magmatism of the EC is observed. In the EC, primitive volcanic rocks of various petrographic and geochemical compositions with ages of <1 Ma have been found, including high-Mg andesite (HMA) (Fig. 1b). Previous studies have reported finding HMA in a variety of specific and temporal tectonic settings, including the subduction of a young slab and asthenospheric injection around the slab edge^3, 13, 14^, and the initiation of subduction of an oceanic plate^15^. The corresponding mechanisms involve slab melting^16^, interaction between slab melt and mantle peridotite^17^, and hydrous mantle melting at low pressure^18, 19^, suggesting a relatively high water content or high temperature condition. In addition, some of the HMA and other mantle-derived primitive magmas contain high-Ni (>3000 ppm) olivine (Ol), which requires a specific source composition or mineralogy such as high-Ni mantle^20^ or a pyroxene-rich source^21–24^.

This study found ultra-high-Ni Ol (up to 6300 ppm Ni with Fo_90_) in the HMA within the EC area. This is the highest value recorded in arc lavas to date^23–25^. A primitive basalt containing moderate–high-Ni Ol (~2900 ppm Ni, Fo_90_) also erupted in a narrow ~60-km N–S along-arc segment nearly simultaneously with high-Mg basalt and high-Al basalt. Determining the genetic conditions (P-T) that could produce such diversity within limited time and space is crucial for understanding the unique tectonic settings and the thermal structure and flow of the mantle dynamics of both the northern edge of the Pacific Plate and the subduction of the extension of the Emperor Seamount Chain^5^ beneath the EC area. Based on the genetic conditions of the HMA, primitive basalt, and ultra-high-Ni Ol, the mechanisms responsible for the enigmatic forearc magmatism are discussed, with consideration of the specific and temporal conditions associated with seamount subduction.

## Results: petrological variability of EC lavas

The K-Ar and ^40^Ar/^39^Ar dating indicated that the EC lavas erupted during the Middle to Late Pleistocene (0.73–0.12 Ma) (Supplementary Table S1), which is in agreement with previous results based on geomorphology^9^. Consequently, the tectonic configuration (e.g., the positions of the arc, trench, fracture zones, and edge of the Pacific Plate) when the EC lavas erupted was similar to the present-day tectonic setting established at ~2 Ma^26^.

**Figure 2:**
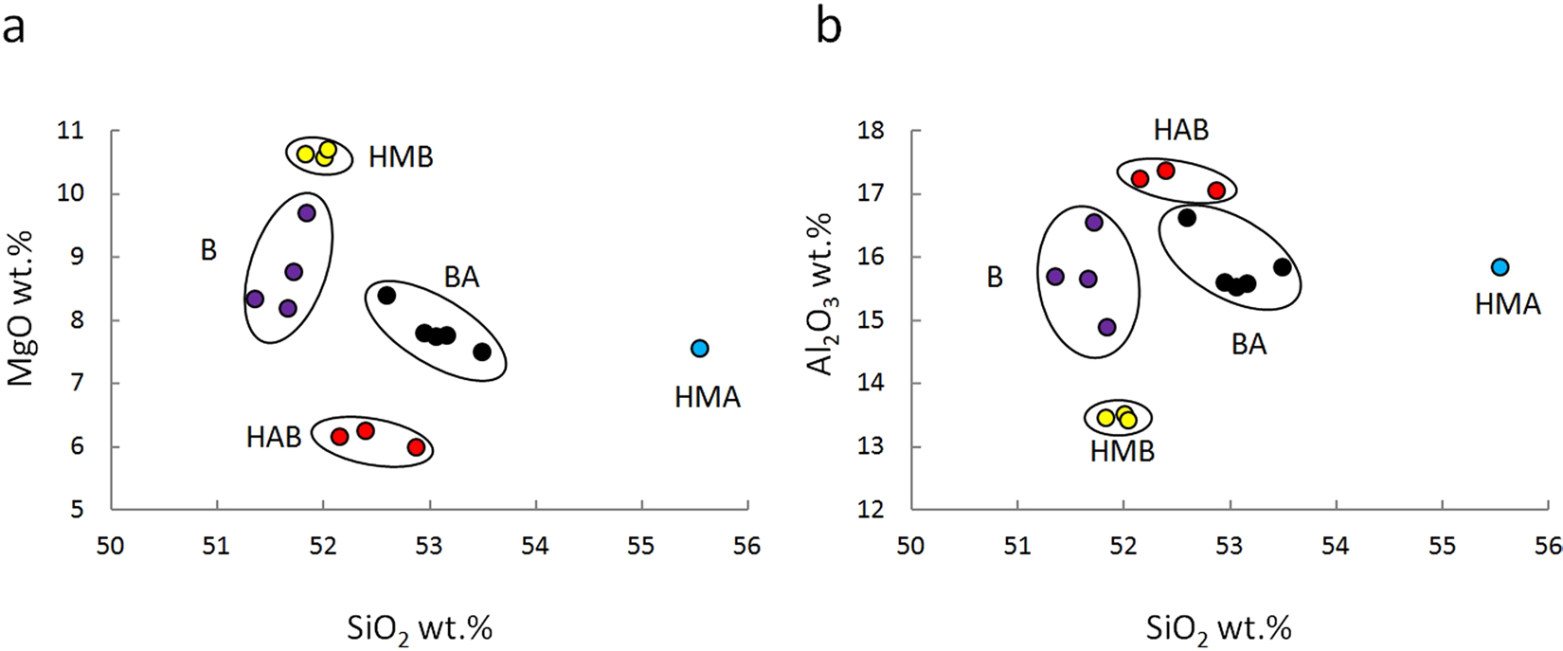
Harker diagrams of the EC lavas. (**a**) SiO_2_ vs. MgO. (**b**) SiO_2_ vs. Al_2_O_3_. Rock type classifications: high-Al basalt (HAB/red), high-Mg basalt (HMB/yellow), high-Mg andesite (HMA/blue), basalt (B/purple), and basaltic andesite (BA/black).

The EC lavas show a common phenocryst assemblage: Ol, clinopyroxene (Cpx), plagioclase (Pl), spinel (Sp) and ±magnetite; however, their textures, modal mineral compositions, and bulk rock major and trace compositions are different (Supplementary Table S2). Based on the SiO_2_, MgO, and Al_2_O_3_ content-, the EC lavas can be classified into five rock types: high-Al basalt (HAB), high-Mg basalt (HMB), high-Mg andesite (HMA), basalt (B), and basaltic andesite (BA) (Fig. 2). It should be noted that, here, rocks with MgO > 6 wt.% and SiO_2_ > 54 wt.% are classified as HMA^27, 28^, and that the nomenclature of other rock types is based on ref. 29. The EC lavas exhibit undifferentiated characteristics with high MgO contents (6–11 wt.%), high Ni contents (39–176 ppm), and low FeO*/MgO (<1), except for the HAB (FeO*/MgO = 1.4). In addition to these undifferentiated characteristics, the major and trace elements show compositional variation with temporal evolutionary trends; with time, the SiO_2_ and Ni contents broadly decrease, whereas the MgO and FeO* contents broadly increase (Supplementary Fig. S1), indicating the effects of various mantle processes and their temporal changes corresponding to the distinct compositional variation from high-Mg andesite to primitive basalts.

**Figure 3:**
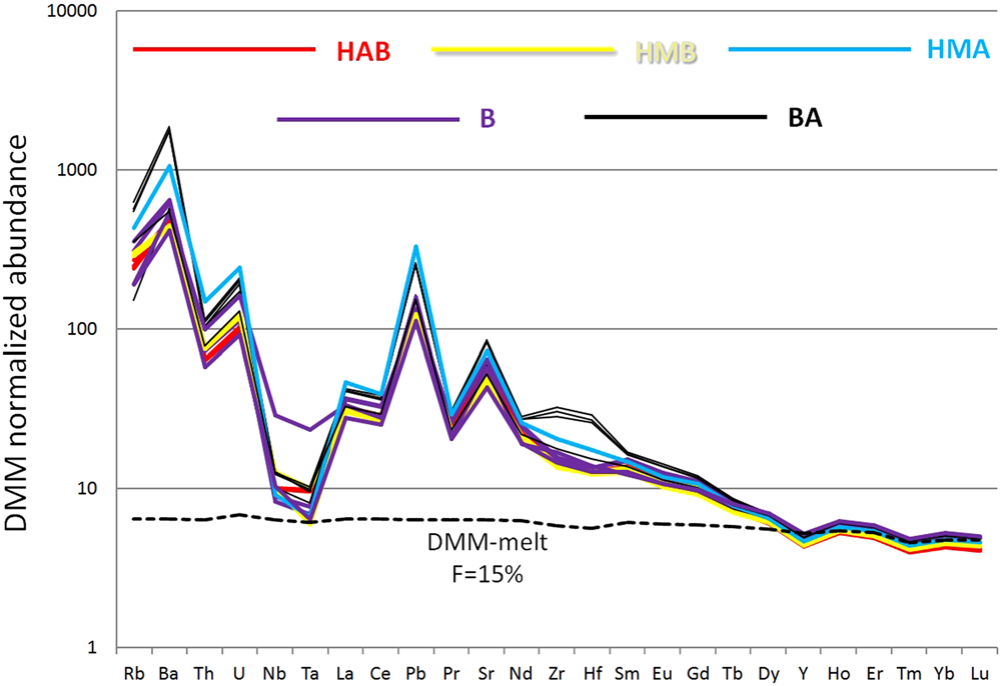
Depleted MORB mantle (DMM)-normalized trace element spidergram for the EC lavas. Colors represent each rock type as identified in Fig. 2. Dashed line represents anhydrous DMM melt with 15% degree of melting at 1.5 GPa^30^.

In the trace element spidergram, all EC lavas show a typical arc signature with relative LILE enrichment and HFSE depletion (Fig. 3). All HREEs converge to a uniform trend that is reproduced remarkably well by melting of anhydrous depleted MORB mantle (DMM)^30^. However, the fluid-mobile elements show some variability, which suggests variable contributions of the amount of the slab-derived component (fluid addition and/or melt). According to a quantitative inversion model for trace element composition^31^, the observed compositions of the HMA and the primitive basalt are best explained by 10–11% and 14% melting of a DMM metasomatized by 0.4 wt.% slab-derived fluid, respectively (Supplementary Table S3).

**Figure 4:**
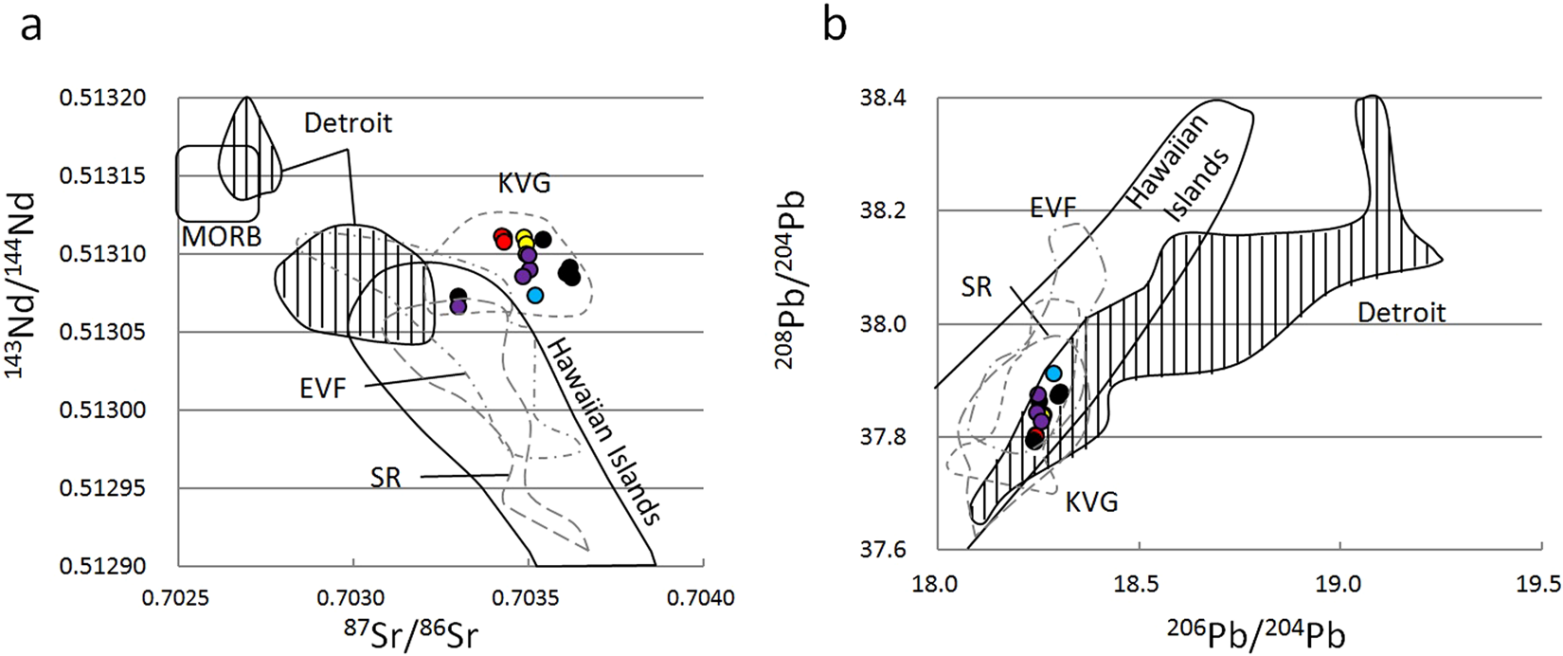
Sr–Nd–Pb isotopic compositions of the EC lavas. **(a)** ^87^Sr/^86^Sr vs. ^143^Nd/^144^Nd and **(b)** ^206^Pb/^204^Pb vs. ^208^Pb/^204^Pb compared with the Detroit Seamount^49, 53^ and Hawaiian Islands (GEOROC geochemical database: http://georoc.mpch-mainz.gwdg.de), MORB^78^, and previously reported compositions for EVF, KVG, and the Sredinny Range (SR) in Kamchatka^7^. Colors represent each rock type as identified in Fig. 2.

The individual rock types show distinctly different Sr–Nd–Pb isotopic compositions, although the overall range is narrow and depleted (^143^Nd/^144^Nd = 0.51307 to 0.51311 and ^87^Sr/^86^Sr = 0.70330 to 0.70362), which indicates minimal crustal contamination (Supplementary Table S4). In the ^87^Sr/^86^Sr–^143^Nd/^144^Nd and ^206^Pb/^204^Pb–^208^Pb/^204^Pb diagrams, the EC lavas are plotted close to or overlapping the KVG lavas and the Detroit Seamount, showing a relatively small range compared with the EVF and the Sredinny Range (Fig. 4).

**Figure 5:**
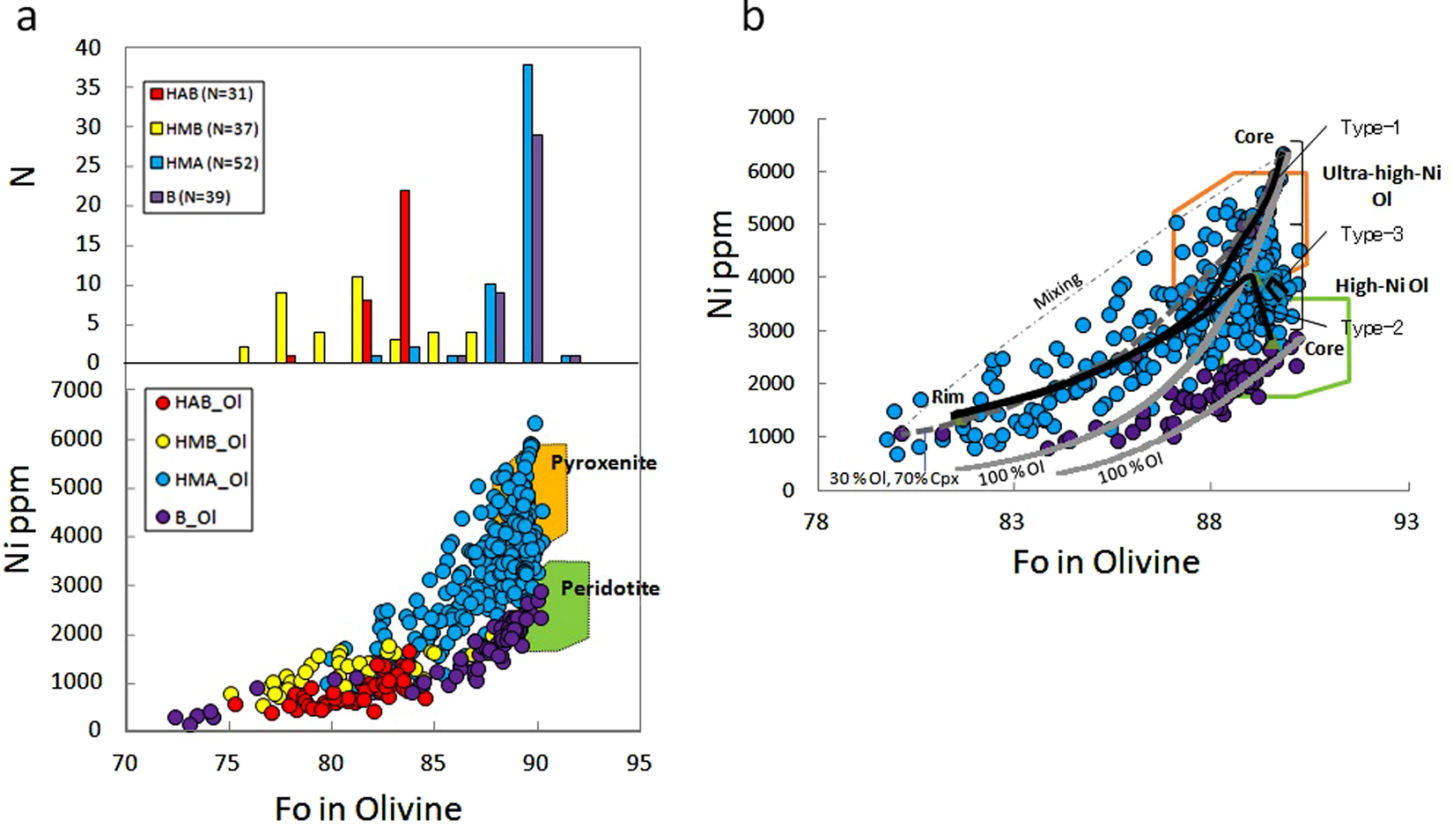
Ni abundance of olivine in the EC lavas. (**a**) Olivine of HAB, HMB, HMA, and B. The upper histogram shows the Fo composition of these cores. Colors represent each rock type as identified in Fig. 2. Green field and ochre field indicate compositional fields of Ol that crystallizes from melts in equilibrium with mantle peridotite and pyroxenites respectively^23, 24^. (**b**) Ni vs. Fo diagram illustrating linear profile of HMA_Ol with B_Ol. Black lines indicate three types of zonal structure from the core to the rim (Type-1, -2, -3) of HMA_Ol. Type-1 trend reflects ultra-high-Ni Ol explained by fractional crystallization (in 0.1% steps) of Ol and Cpx with proportions of ~3:7 (gray dashed line) from ultra-high-Ni melt at equilibrium with ultra-high-Ni olivine (~6300 ppm Ni)^20, 79, 80^. This could not be explained by mixing between a mafic component with high Ni content and a felsic component, as observed in the HMB_Ol. Complementary evidence for this model is that the HMA_Ol has no reverse zoning of Fo content and there is no HMA_Ol with low Fo and Ni contents derived from derivate melt. On the other hand, the trend of B_Ol can be explained by fractional crystallization of Ol (gray line) at Fo_85–90_. Areas surrounded by a green and ochre borders indicate compositional fields of Ol that crystallizes from melts in equilibrium with mantle peridotite and pyroxenites respectively^23, 24^.

The Ol phenocrysts also exhibit different compositional ranges for individual rock types (Fig. 5a, Supplementary Table S5). The primitive basalt contains Ol phenocrysts of predominantly Fo_88–90_ and 2500–2900 ppm Ni (B_Ol, purple circles in Fig. 5), which fall within the compositional range of Ol that crystallizes from melts in equilibrium with mantle peridotite^23, 24^ (Fig. 5a). The downward convex trend of the B_Ol from relatively high to low Fo and Ni contents can be explained by Ol fractional crystallization (Fig. 5b).

The HMA also contains Ol phenocrysts of predominantly Fo_88–90_ and it can be in equilibrium with mantle peridotite rocks (HMA_Ol, blue circles in Fig. 5). However, the HMA_Ol contains more Ni (maximum: ~6300 ppm) than the B_Ol (maximum: ~2900 ppm). This Ni content is the highest value reported thus far in arc lavas^23–25^, which broadly fall within the compositional range of Ol that crystallizes from melts in equilibrium with pyroxenite^23, 24^, as shown in Fig. 5a. The HMA_Ol has a wide range of Ni content (2500–6300 ppm) for a small range of Fo_88–90_, and three types of zonal structure from the core to the rim (Fig. 5b). These features suggest melting and crystallization processes distinct from those of the primitive basalt produced by melting of a peridotitic source and subsequent Ol-dominant crystallization. For instance, the three types of zonal structure shown in Fig. 5b might correspond to simultaneous crystallization of Ol and Cpx in the proportion of ~3:7 from an ultra-high-Ni melt (Type-1), incorporation of Ol with a composition identical to that of B_Ol into the ultra-high-Ni melt and subsequent overgrowth (Type-2), and equilibrium crystallization to produce a nearly constant composition from the core to the rim (Type-3). These features reflect a series of simultaneous but distinct processes within the source region of the HMA. In any case, the B_Ol cannot be derived from the HMA by crystallization of Ol and Cpx in various proportions, and vice versa. Therefore, heterogeneous mantle sources are required to explain the HMA and B.

**Figure 6:**
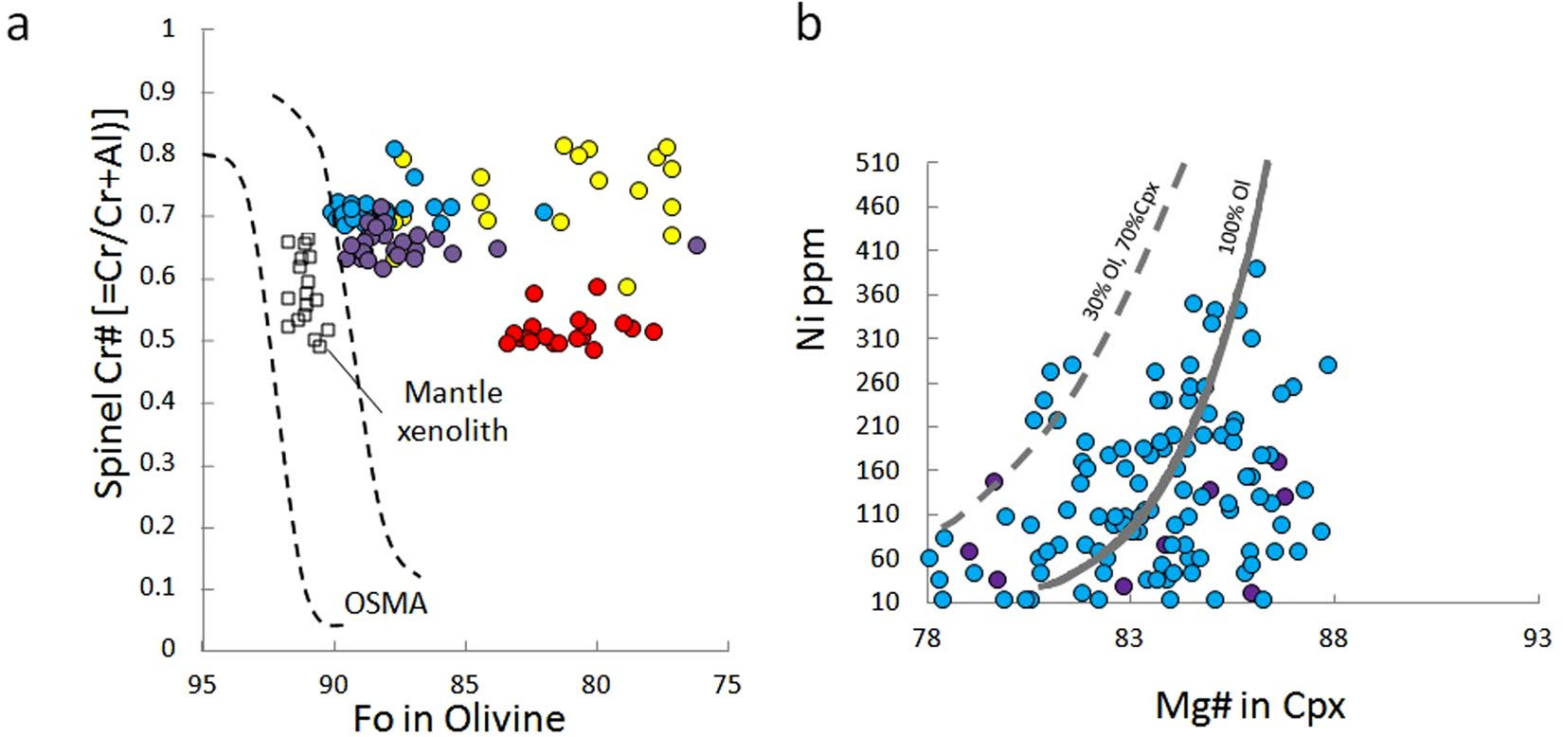
Mineral compositions of the EC lavas. (**a**) Cr# in Sp vs. Fo diagram. Mantle array (OSMA)^29^ shown by two dashed lines. (**b**) Ni abundance of Cpx in the HMA and B. Gray dashed line indicates fractional crystallization trend of Ol and Cpx and solid gray line indicates olivine fractional crystallization trend from primitive melt, as in Fig. 5b. Colors represent each rock type as identified in Fig. 2.

The Cpx phenocrysts in the HMA have relatively high Ni contents (>200 ppm Ni) for relatively low Mg values (<83). They are plotted on a trend indicating simultaneous crystallization of Ol and Cpx (Fig. 6b), which suggests indirectly that the Cpx formed during an early stage of crystallization at relatively high pressure. The presence of pargasitic hornblende in the HMA, which has broken down into small mineral aggregates and exhibits a composition similar to that stable in hydrous peridotite^32^, also suggests crystallization at relatively high pressure and breakdown by subsequent decompression. Therefore, the HMA appears to have undergone various crystallization processes in the mantle and transported materials during its ascent.

Ol phenocrysts in the HMB (HMB_Ol, yellow circles in Fig. 5a) and HAB (HAB_Ol, red circles in Fig. 5a) represent suites that are more differentiated (Fo_75–88_ and Fo_75–85_, respectively, with Ni < 2000 ppm). The downward convex trend of the HAB_Ol can be explained by Ol fractional crystallization, whereas the HMB_Ol shows a linear trend (Fig. 5a), exhibiting a broad frequency distribution of the core compositions (Fig. 5a). The linear trend of the HMB_Ol can be reproduced by mixing between a mafic component with high Ni-content (1988 ppm Ni, Fo_87.8_) and a silicic component (542 ppm Ni, Fo_76.6_). However, it is difficult to identify the primary melts (HMA-type or B-type) that are related to the HAB and HMB because of their relatively differentiated characteristics.

Regardless of rock type, the Ol phenocrysts contain Cr-spinel inclusions. The frequency distributions of Cr content for the B_Ol, HMA_Ol, and HMB_Ol show peaks at 44, 48, and 26 wt.% Cr_2_O_3_, respectively, whereas HMB_B exhibits a broad range (10–44 wt.% Cr_2_O_3_). In the Fo vs. Cr/(Cr + Al)_spinel_ (=Cr#) diagram, which provides a measure of the depletion of the source mantle^33^, the HMA and B show high values of Cr# (0.60–0.75), similar to that of the most depleted mantle reported from the mantle xenoliths in Kamchatka^34, 35^ (Fig. 6a).

## Discussion

### Origin of the forearc EC magmatism

Based on their bulk compositions (Figs 2–4) and the Fo–Cr# relationship (Fig. 6a), it is considered that the EC lavas are products of the melting of a DMM-like depleted mantle that was metasomatized by slab-derived fluids. The age of the Pacific Plate near the Kamchatka Peninsula is 90–100 Ma^11^, and a subducting slab of this age generally does not dehydrate at shallow depths beneath the forearc region^12^. According to recent thermal modeling of subduction zone processes, including the generation and movement of slab-derived fluids^36–38^, the slab surface temperature beneath the EC (depth: 50–80 km) is estimated at <400 °C^36–38^. Theoretically, at such temperatures, slab-derived fluid should not be supplied to a mantle wedge because of the low slab dehydration rate and the almost complete absorption of the slab-derived fluid by serpentinization just above the slab. To deliver an appreciable amount of slab-derived fluid to the mantle wedge beneath the EC area, it would be necessary to have a relatively warm subducting slab and conditions that allow the slab-derived fluid to ascend locally without absorption by serpentinization. Considering the unique tectonic setting of the EC area, there are two possibilities for warming of the subducting slab: thermal influence from the subduction of a seamount^5^ or heating by asthenospheric injection around the slab edge^3^.

**Figure 7:**
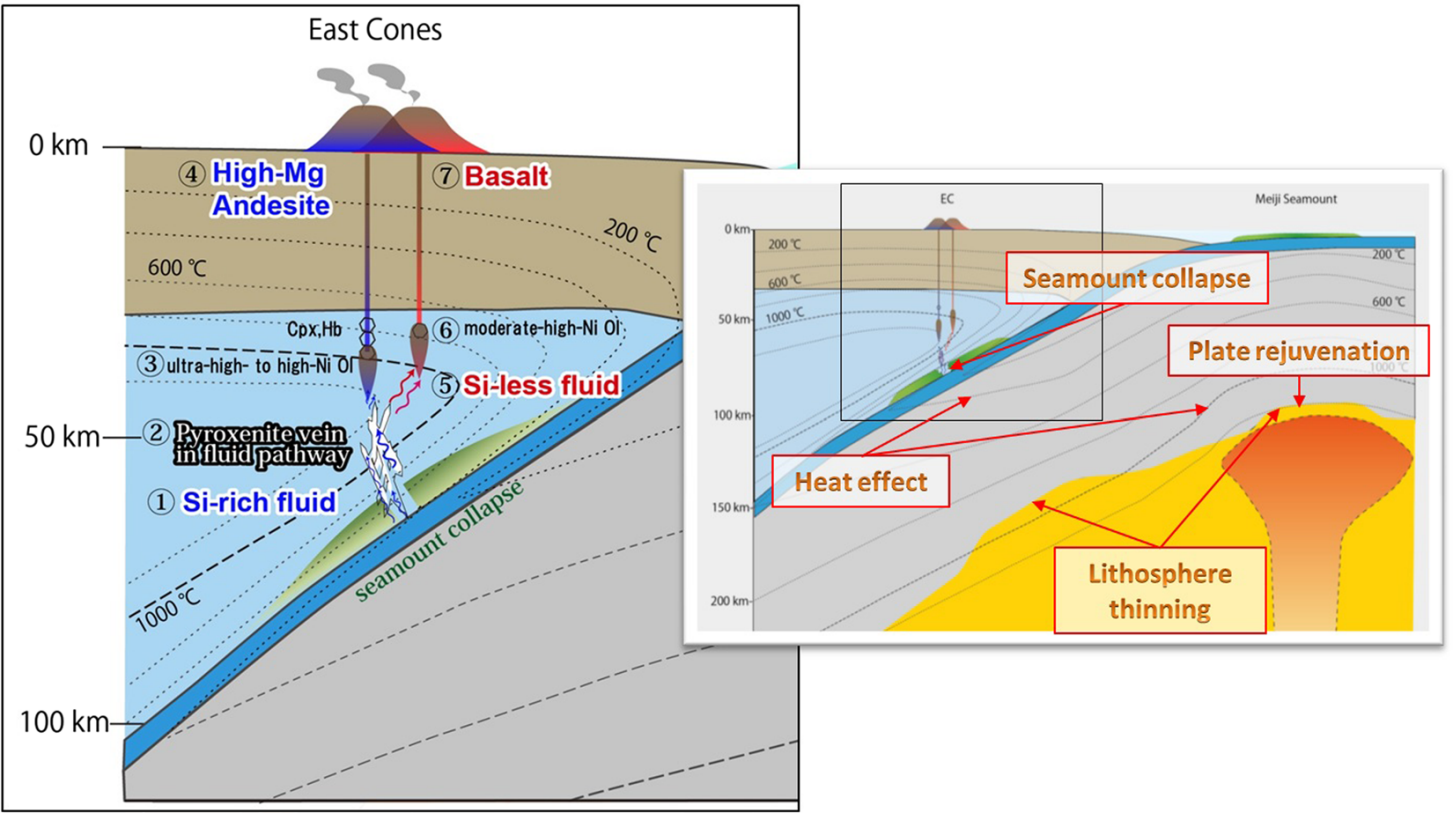
Cross section of northern subduction zone of the Kamchatka Peninsula and schematic model of EC magma generation. Right figure shows overall cross-sectional view of the subduction zone and it represents why the subducted seamount is the cause of efficient slab dehydration just beneath the EC area (<80 km), via the following effects: thinning of the lithosphere^5^, plate rejuvenation from a mantle plume^40, 41^, and formation of fluid pathway along cracks formed by the collapse of the subducted seamount. Left figure (black frame part in right figure) focuses on the mantle wedge beneath the EC and it represents the genesis of EC lavas. Based on these effects, ① silica-enriched slab fluids are dehydrated from the subducted seamount and ② form pyroxenite veins in the mantle wedge, which are formed locally along fluid pathways. Its melting would ③ generate ultra-high-Ni melt (HMA) and ④ crystallize ultra-high-Ni olivine (~6300 ppm Ni) at the initial stage of crystallization in the mantle. Cpx also starts to crystallize from the initial stage and pargasitic hornblende (Hb) breakdown by subsequent decompression. For subsequent stages, ⑤ the residual Si-less fluid would cause flux melting of peridotite to produce basalt with moderate–high-Ni olivine (~2900 ppm Ni) ⑥ and ⑦. The black dashed lines indicate temperature contours from 200 to 1200 °C with a contour of 200 °C intervals. The seamounts and EC are not drawn to scale.

The offset of the volcanic front at around 55°N from the EVF to the CKD corresponds to a shallowing of the slab dip angle from 55° to 35°, which is considered to be a consequence of the subduction of the Emperor Seamount Chain^2^. Ophiolites exposed on the Kamchatsky Peninsula^39^ preserve old (120–93 Ma) products of a hotspot that exhibit geochemical affinities to an older part of the Emperor Seamount Chain, including the Detroit and Meiji seamounts. The oceanic lithosphere around the Detroit and Meiji seamounts is thinner (by 10%–30%) in comparison with the surrounding region^5^, which is explained by delayed thickening of the lithosphere below the Meiji–Hawaiian hotspot associated with small-scale convection triggered by plume–lithosphere interaction at ~80 Ma^5^. In addition, moderate heating by another mantle plume (i.e., plate thermal rejuvenation without melt supply) is suggested based on heat-flow measurements and a low-velocity zone extending from a depth of ~900 km beneath the current position of the Meiji Seamount^40, 41^. If such a seamount were subducted, a thermal effect similar to that associated with the subduction of a young slab might occur locally, where the slab surface temperature could reach 600 °C. This would cause efficient slab dehydration and breakdown of serpentinite just beneath the EC area^12, 42^. The collapse of subducted seamounts, which is suggested based on geological and seismological studies^43, 44^, would have promoted fluid ascent by forming cracks beneath the EC area (Fig. 7).

### Metasomatism by Si-rich fluid from subducted seamount

When such fluid is supplied to an overlying mantle wedge, pyroxenite veins are formed locally along fluid pathways and its melting would generate a high-Ni melt^23, 24^. Under the pressure (~2 GPa) and temperature (~600 °C) conditions beneath the EC area, the slab-derived fluid would dissolve significant amounts of silica^45^, and the increase in orthopyroxene (Opx) modal composition of the metasomatized mantle would significantly lower the bulk partition coefficient of Ni (D_Ni_^rock/melt^) compared with that of peridotite^46–48^, efficiently partitioning Ni into the melt. If approximately half the amount of Ol in peridotite is reacted to form Opx, D_Ni_^rock/melt^ is reduced to ~7/10 compared with the original peridotite source, and the calculated Ni content of the melt would be 90–520 ppm, including the uncertainty in the partition coefficients (Supplementary Table S3). This value might satisfy the necessary conditions for producing an ultra-high-Ni melt (466–612 ppm Ni) from which ultra-high-Ni (up to 6300 ppm) Ol could crystallize, provided that approximately 8 wt.% Ol is fractionated from the primary melt during the initial stages of crystallization (Supplementary Table S3). This pyroxenization would occur in various proportions along the fluid pathways, e.g., the closer to the fluid source, the greater the pyroxenization, and the generated melt would show variable Ni content with a nearly constant Mg#, which explains the characteristics of the HMA_Ol shown in Fig. 5.

The Detroit Seamount^49^ yields picritic basalts that contain significantly more Ni (~1300 ppm Ni) than either oceanic sediment (~20 ppm Ni)^50^ or AOC/MORB (~92 ppm Ni)^51^. If such rocks were subducted and dehydrated, the slab-derived fluid would become enriched in Ni and silica, enhancing the possibility of producing a high-Ni melt. In addition, a quantitative inversion model of incompatible trace elements has indicated that the Detroit Seamount, as a source of slab-derived fluid, explains the EC lava compositions better compared with cases using the composition of Pacific Plate AOC^31, 52^. These examples of geochemical evidence support the suggestion that a subducted seamount contributed to the generation of the EC magmas and that it probably had an influence on all the lavas along the entire northern Kamchatka traverse, as was suggested earlier^6–8^. The Sr–Nd isotopic compositions of the Detroit Seamount exhibit the most depleted characteristics within the Emperor Seamount Chain, reflecting a refractory component of the plume that was sampled only at high degrees of melting. The reason for the higher degree of melting is not because of the high potential temperature of the mantle but because of the extensive adiabatic melting to a shallower depth beneath the relatively thin oceanic crust when the Hawaiian plume was near a spreading center at ~80 Ma^49, 53^. The subducted seamount that contributed to the formation of the EC magmas is expected to have had the same geochemical nature as the Detroit Seamount. The slightly enriched ^87^Sr/^86^Sr composition of the EC compared with the Detroit Seamount could be explained if the slab fluid that metasomatized the source mantle involved a small amount (~1%) of a sediment-derived component^6^. It is emphasized that the EC lavas are not hotspot magmas, but are arc magmas affected by the subducted seamount, which is supposed of hotspot origin, via slab-derived fluids, as discussed above.

A model for the heating of the slab by asthenospheric injection around the slab edge is not supported for the following reasons. The trace element inversion model indicates the degree of melting of the EC lavas was 10%–14% and that the water content of the mantle wedge was 0.4% at 1–2 GPa, indicating a melting temperature of 1150–1250 °C^12, 54^. This temperature is lower than a potential temperature of hotspots, and even lower than the average mantle potential temperature (~1300 °C^55^), but it is similar to that beneath the forearc area in the NE Japan arc where the Pacific Plate of the same age is subducted^37^. Because the mantle beneath the forearc area constitutes a narrow wedge intercalated between the arc crust and the subducting slab, it is difficult for asthenospheric injection to intrude and heat the slab surface beneath the forearc region, including the EC area^3^. This is unlike the Shiveluch Volcano located on the slab edge, where slab melting is considered attributable to hot asthenospheric flow^3, 25^.

### Compositional variability and evolution of source mantle

These thermal structures and fluid processes associated with a warm slab but a relatively low-temperature mantle wedge could explain the petrological variability of the EC lavas and the mineralogical heterogeneity of the HMA (Fig. 7). The three types of HMA_Ol (Fig. 5b) indicate several isolated melt pockets and/or veins at the initial stage of crystallization in the mantle, each being derived from different degrees of pyroxenization along the fluid pathways. At the initial stage, Cpx was crystallized with Ol from an ultra-high-Ni melt, which would have formed the Type-1 and -2 trends of the HMA_Ol, as shown in Fig. 5b.

Generally, an Ol liquidus field expands as a primary melt ascends from the region of mantle melting. In turn, simultaneous crystallization of Cpx and Ol from the primary magma requires a condition close to the melting condition, under which compositions evolve with lower CaO, SiO_2_, and MnO and higher FeO and NiO^20^ compared with those derived by Ol-only fractionation. This is consistent with observations, e.g., the composition of ultra-high-Ni Ol exhibits not only high Ni contents, but also elevated Fe/Mn values (~100 for HMA_Ol, ~86 for B_Ol) and depleted Ca (~336 ppm for HMA_Ol, ~650 ppm for B_Ol) and MnO contents (~805 ppm for HMA_Ol, ~895 ppm for B_Ol). Efficient cooling of magma close to the melting condition is needed for the fractionation of Cpx during the initial stage, as illustrated in Fig. 7. The presence of pargasitic hornblende in the HMA also supports a crystallization condition close to that of hydrous peridotite solidus (<2.5 GPa and <1250 °C)^12, 54^. Pargasite crystallized during the early cooling stage, possibly within a melt pocket or along the vein wall, and it was later mixed with magmas from other melt pockets with different zoning types of Ol.

Within the short period of activity of the EC, the distinct compositional variation occurred with a systematic temporal evolution from the HMA to primitive basalts (Supplementary Table S1). At the initial stage with the HMA, fluid pathways had not developed extensively and a significant proportion of the slab-derived fluid would have reacted to form pyroxenite in varying degrees along the pathway, as recorded in three types of HMA_Ol phenocrysts (Fig. 5b). Once melting had occurred and the HMA magma was extracted, the pyroxenite source became exhausted. Subsequently, the residual fluid that had already precipitated silica would have caused flux melting of the peridotite to produce the primitive basalt. This source mantle evolution is supported by the temporal evolution in terms of the major and trace element compositions (Supplementary Fig. S1); e.g., pyroxenites might produce high-Mg andesitic melts with higher Ni compared with peridotites^56^. The fluid network would have developed upward, assisting the smooth ascent of the melt, which would have crystallized mostly Ol without developing isolated melt pockets. Such temporal evolution suggests a limited supply of slab-derived fluid, which is consistent with a local subducted seamount as the source of the fluid. Therefore, the HMA, including the ultra-high-Ni Ol, and subsequent magmatism are considered to reflect a series of products that originated from varying degrees and types of interaction between the mantle wedge and slab-derived fluid supplied from a subducted seamount over limited time and space (Fig. 7).

Such a suite of local and temporal magmatism triggered by seamount subduction could occur in other subduction zones. For example, the Galapagos track on the Cocos Plate is subducting beneath central Costa Rica, where geochemical contribution from the subducted seamount is suggested based on isotopic signatures of the volcanic rocks and Ol-hosted melt inclusions in central Costa Rica^57, 58^. High-Ni Ol (~4300 ppm Ni, ~Fo_90_) can be seen in the primitive basaltic andesites of the Irazu Volcano, which is located to the northeast of the seamount track^59^. On the other hand, the northwestern part of the Cocos Plate has no apparent seamount edifices, and it is subducting beneath the Quaternary central Mexican Volcanic Belt (MVB) located to the northwest of Costa Rica. However, high-Mg# basalts and andesites occur in the MVB and they contain high-Ni Ol (~5400 ppm Ni, ~Fo_90_)^23, 24^. Reference 60 argued that these high-Ni Ols were derived from pyroxenites formed by infiltration of multiple silicic components from the subducting slab. i.e., the subducted coastal and offshore granodiorites, subducted AOC, and a subducted seamount component. Although the MVB could have been affected to some extent by the subducted seamount, the simultaneous occurrence of the HMA with high-Ni Ol and other types of primitive lava might not uniquely indicate seamount subduction; therefore, other components and models need to be examined. However, seamount subduction is at least one of the important factors that we should examine for constraining past tectonic environments under unclear settings where such volcanic assemblages occur.

## Method

### Whole-rock geochemical analyses

Weathered parts were removed for the bulk analyses of major elements, trace elements, and isotope ratios. The samples were crushed coarsely (size: 2–4 cm) and cleaned using an ultrasonic bath with deionized water (DIW). Samples were then crushed by hand into finer grains using a tungsten mill. Finally, these fine-grained samples were powdered using a quartz ball mill (Fritsch; Planetary mill pulversette 5).

### Whole-rock major elements analyses

Major elements were analyzed by an X-ray fluorescence spectrometer (Rigaku; RIX-2100) using the glass bead method with a sample:flux ratio of 1:10^61^. The flux (MERC; Spectromelt A10) was anhydrous lithium tetraborate, which was evaporated completely by heating (4 h/650 °C) in an electric muffle furnace in advance. The samples were also evaporated by heating (12 h/900 °C) in an electric muffle furnace in advance. Both were weighed accurately with an electronic balance and mixed together with agate mortar. The mixture was then made into a glass bead using a fuse-sampler machine (Rigaku; sample pretreatment device).

### Whole-rock trace element analyses

The abundance of trace elements was determined using the acid digestion method^62^. Powdered splits were dissolved by acid digestion (HClO_4_ and HF) and heated for three days. The decomposed sample was evaporated stepwise until dry. Subsequently, aqua was added to the residue, which was heated for 12 h, and then evaporated. The residue was dissolved with HNO_3_ and the solution diluted to 1000 times by mass. The diluted solutions were analyzed using inductively coupled mass spectrometry (ICP-MS) (Thermo; X Series II).

### Whole rock Sr–Nd–Pb Isotopes

Sr, Nd, and Pb isotope analyses were conducted at Japan Agency for Marine-Earth Science and Technology (JAMSTEC). The whole rock powder samples for Pb isotope analysis were leached by 1 M HCl at room temperature. The analytical procedure used for chemical separation and mass spectrometry for the Sr, Nd, and Pb isotope determinations is outlined in refs 63–68. Total procedural blanks for Sr, Nd, and Pb during the measurement period were less than 177 pg, 5, and 10 pg, respectively. The Sr and Nd isotope ratios were measured by thermal ionization mass spectrometry (TIMS) (Triton TI: Thermo-Finnigan). The Sr and Nd isotope ratios were normalized to ^86^Sr/^88^Sr = 0.1194 and ^146^Nd/^144^Nd = 0.7219, respectively, to correct for mass fractionation. The mean ^87^Sr/^86^Sr value in the NIST SRM987 was 0.710220 (±0.000012; 2σ, n = 13) and the mean ^143^Nd/^144^Nd value in the JNdi-1 standard was 0.512098 (±0.000013; 2σ, n = 15). The Pb isotope ratio was determined by a multiple-collector (MC)-ICP-MS (Neptune; Thermo Scientific). Mass fractionation factors for Pb were corrected using Tl as an external standard. Additional mass-dependent interelement fractions were also corrected by applying a standard bracketing method using NIST SRM981 as a standard. The ^206^Pb/^204^Pb, ^207^Pb/^204^Pb, and ^208^Pb/^204^Pb of repeated measurements of NIST SRM981 were 16.9313 ± 0.0008, 15.4851 ± 0.0009, and 36.6780 ± 0.0029 (2σ, n = 53), respectively. The Pb isotope data are presented after recalibration using the NIST SRM981 values of ^206^Pb/^204^Pb = 16.9416, ^207^Pb/^204^Pb = 15.5000, and ^208^Pb/^204^Pb = 36.7262 reported by ref. 68.

### Olivine composition analyses

After crushing lava blocks (100 g) using a laboratory fragmenter (SELFRAG) installed at JAMSTEC, we picked olivine phenocrysts under a binocular stereo microscope. Olivine phenocrysts were set in resin (Technovit No. 4071) and polished to expose the olivine surface. Major element analyses of Olivine were performed at the Earth–Life Science Institute using an Electron Probe Micro Analyzer (EPMA; JAX-8800M (JEOL)) using a 15-kV accelerating voltage and 1.2 × 10^−8^-A beam current with 1-μm beam size. The ZAF correction was then applied.

### K–Ar and ^40^Ar/^39^Ar age analyses

Samples for K**–**Ar dating were washed with DIW. After drying, the samples were crushed coarsely using a tungsten mortar mill and then filtered with sieves (#32–64) to 250–500 μm. The crushed samples were rinsed with DIW in an ultrasonic bath. To avoid excess ^40^Ar, large phenocrysts (>0.5 mm) were removed by hand picking and an isodynamic magnetic separator.

For potassium analysis, the 3–5-g subsamples used for the argon isotope analysis were pulverized further using an agate mortar.

Argon isotopic ratios were determined by a conventional isotope dilution method using an ^38^Ar spike. Argon extraction from a sample was performed using a stainless steel ultra-high vacuum extraction line. Samples of 0.35–0.40-g were wrapped in 10-µm copper foil and fused at 1500 °C using a double-vacuum tantalum resistance furnace following by bakeout at 120 °C for 72 h. The ^38^Ar spike was removed from the reservoir tank using a pipette valve and mixed with sample gas during fusion of the sample. Purification of the sample gas was achieved with two SAES Getters NP-10 Sorb-Ac pumps and one zirconium and titanium plate getter. Argon isotopes were measured on a VG Isotopes 1200 C mass spectrometer equipped with a Nier-type ion source and a single Faraday collector. Argon isotopes of an air standard were analyzed once or twice daily for mass discrimination correction.

Potassium contents were determined for 200-mg samples by flame emission spectrometry, using peak integration and the lithium internal standard method^69^. Error for the potassium analysis was estimated at 0.5% based on the replicate analyses of reference materials^69^.

^40^Ar/^39^Ar Age determinations were made using the ^40^Ar/^39^Ar geochronology facility at the Geological Survey of Japan/AIST following the analytical procedure described in ref. 70.

### EC-magma generation modeling

The inversion model for estimating the melting condition of the EC lavas^31^ consisted of a dehydration process from the slab and a melting process in the mantle wedge. The composition of the slab component (C_SM) was based on the average composition of the Detroit Seamount (99.8 ppm Ni), which was estimated from the composition of each layer and the thickness ratios of each of the drilling cores^49, 53^. Slab-derived fluid composition (C_f_SM) was estimated from the dehydration rate (s; ~5%) and partition coefficient D^fluid/rock^ (D_SM)^71^. In the melting process, source mantle (C_s) metasomatized by the slab-derived fluid was flux-melting. The original mantle lithology was assumed peridotite or pyroxenite and its bulk composition was DMM^72^. However, the Ni contents considered the variations estimated from the peridotite xenoliths in Kamchatka^34^. The transformation of mantle olivine to orthopyroxene (“reaction orthopyroxene”) by siliceous slab-derived fluid formed the pyroxenite, which assumed that 10–30 vol.% forsteritic olivine was destroyed (equivalent to destroying 17.5–52.6 vol.% of peridotite olivine). The Ni in the pyroxenite was diluted by the addition of SiO_2_ (~8% dilution)^23^. The melt composition (C_melt_calc) was calculated using a formula for modal batch melting. The bulk partition coefficient D^melt/rock^ was based on a theoretical value dependent on the pressure for peridotite^30^ and an integrated value of the mineral/melt partition coefficient^31^ and modal value for pyroxenite. After generating the melt, it ascended quickly while maintaining non-equilibrium conditions with the wall rock and retaining the initial composition of the eruption. Overall, 25 trace elements and 2 major elements (K, Ti) were calculated, and the melting condition (H_2_O content, degree of melting, pressure*) was estimated based on comparison with a measured value (misfit = log_10_ (C_calc/C_obs)) and its validity (*only for peridotite) (Supplementary Table S3).

## Electronic supplementary material

Supplementary information
