# Supplementary material for: Genesis of ultra-high-Ni olivine in high-Mg andesite lava triggered by seamount subduction
Source: Sci Rep. 2017 Sep 14;7:11515. doi: 10.1038/s41598-017-10276-3 (PMC5599635; doi:10.1038/s41598-017-10276-3)
Supplement: Supplementary file 1 — Supplementary information [file 41598_2017_10276_MOESM1_ESM.pdf]

Supplementary information to the manuscript

Genesis of ultra-high-Ni olivine in high-Mg andesite lava triggered by seamount subduction

Tatsuji Nishizawa[1]*, Hitomi Nakamura[1,2,3], Tatiana Churikova[4], Boris Gordeychik[5], Osamu Ishizuka[6,7], Satoru Haraguchi[2], Takashi Miyazaki[2], Bogdan Stefanov Vaglarov[2], Qing Chang[2], Morihisa Hamada[2], Jun-Ichi Kimura[2], Kenta Ueki[8], Chiaki Toyama[9], Atsushi Nakao[8] , and Hikaru Iwamori[1,2]

[1]Department of Earth and Planetary Sciences, Tokyo Institute of Technology, 2-12-1 Ookayama, Meguro-ku, Tokyo 152-8551, Japan

[2]Department of Solid Earth Geochemistry, Japan Agency for Marine-Earth Science and Technology, 2-15 Natsushima-cho, Yokosuka, Kanagawa 237-0061, Japan

[3]Chiba Institute of Technology, ORCeNG, 2-17-1 Tsudanuma, Narashino, Chiba 275-0016, Japan.

[4]Institute of Volcanology and Seismology, Far East Branch, Russian Academy of Sciences, 9 Piip Boulevard, Petropavlovsk-Kamchatsky 683006, Russia

[5]Institute of Experimental Mineralogy, Russian Academy of Sciences, 4 Academica Osypyana ul., Chernogolovka, Moscow region 142432, Russia

[6]Institute of Earthquake and Volcano Geology, Geological Survey of Japan, AIST, Central 7, 1-1-1 Higashi, Tsukuba, Ibaraki 305-8567, Japan

[7]R & D Center for Ocean Drilling Science, Japan Agency for Marine-Earth Science and Technology, 2-15 Natsushima-cho, Yokosuka, Kanagawa 237-0061, Japan

[8]Earthquake Research Institute, The University of Tokyo, 1-1-1 Yayoi, Bunkyo-ku, Tokyo 113-0032, Japan

[9]Institute of Geology and Geoinformation, Geological Survey of Japan, AIST, Central 7, 1-1-1 Higashi, Tsukuba, Ibaraki 305-8567, Japan

*Corresponding author: Tatsuji Nishizawa, Department of Earth and Planetary Sciences, Tokyo Institute of Technology, 2-12-1 Ookayama, Meguro-ku, Tokyo (Japan). Email: nishizawa.t.ad@m.titech.ac.jp

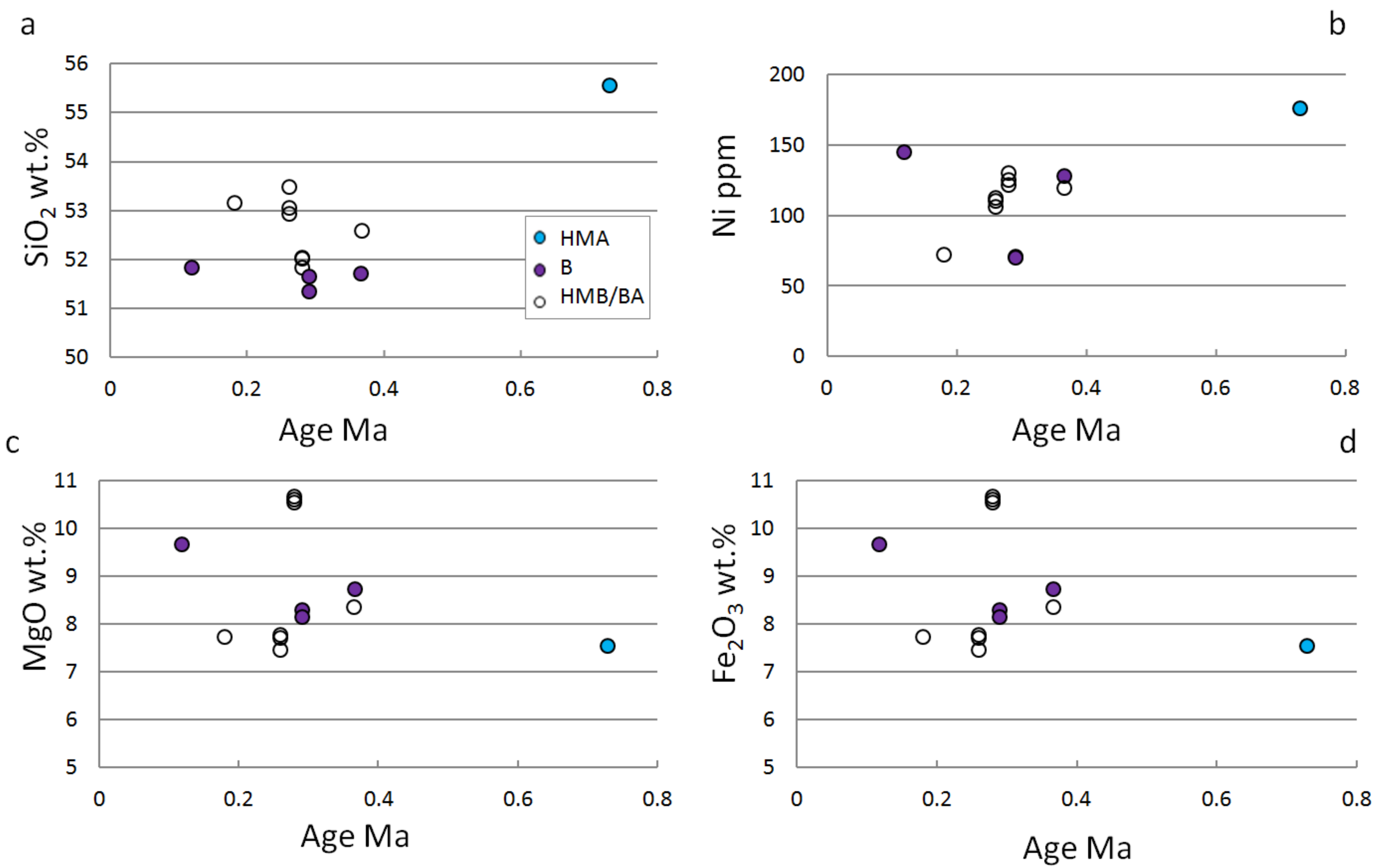

Supplementary Figure S1. Temporal evolution of the compositions with time; the a)SiO_2 and b)Ni contents broadly decrease, whereas the c)MgO and d)Fe_2O_3 contents broadly increase.

Supplementary Table S1. **Summary of K-Ar or ^{40}Ar/^{39}Ar age analyses for EC lavas.**

Sample	Age (±1σ) (Ma)	Integrated age (±1σ)(Ma)	Plateau age (±1σ)(Ma)	Method	K_2O (wt.%)	^{40}Arrad (10^{-6}mlSTP/g)	Atm Ar (%)	^{40}Ar/^{36}Ar intercept	MSWD	Fraction of ^{39}Ar (%)
EC-13-2/4	0.29 ± 0.07	-	-	K-Ar	1.280	0.0118	97.5	-	-	-
EC-13-3/2	0.28 ± 0.03	-	-	K-Ar	1.149	0.0101	93.9	-	-	-
EC-13-4/1	0.26 ± 0.03	-	-	K-Ar	1.943	0.0163	95.7	-	-	-
EC-13-5/2	0.37 ± 0.25	-	-	K-Ar	0.893	0.0106	99.2	-	-	-
EC-13-6/1	-	0.58 ± 0.06	0.73 ± 0.06	^{40}Ar/^{39}Ar	-	-	-	292.7±4.2	0.99	74.2
EC-13-7/1	0.18 ± 0.07	-	-	K-Ar	1.314	0.0077	98.4	-	-	-
EC-13-8/1	0.12 ± 0.44	-	-	K-Ar	0.892	0.0034	99.8	-	-	-

MSWD Means Square Weighted Deviates. Average age is weighted average of the plateau ages from the run.

Supplementary Table S2. **Major and trace element compositions in the EC lavas.**

Cone	EC-1			EC-2		EC-3			EC-4			EC-5		EC-6	EC-7	EC-8	JB-2
Sample	1/1	1/2	1/3	2/1	2/4	3/1	3/2	3/4	4/1	4/2	4/3	5/1	5/2	6/1	7/1	8/1	-
N°	55°42'9.80"	←	←	55°50'22.87'	←	55°48'255"	55°48'17.37'	←	55°51'736"	55°51'44.09'	←	55°54'6.38"	55°54'239"	55°57'57.51'	56° 0'23.09"	56° 6'58.73"	-
E°	161°41'37.77	←	←	161°48'40.49	←	161°55'300"	161°55'14.53	←	161°51'167"	161°51'10.13	←	161°48'6.78"	161°48'044"	161°48'50.59	161°51'34.74	162° 4'40.95'	-
Slab depth (kr	75			68		64			66			67		65	65	52	-
Rock-type	HAB	HAB	HAB	B	B	HMB	HMB	HMB	BA	BA	BA	BA	B	HMA	BA	B	STD
SiO_2 (wt.%)	52.16	52.87	52.40	51.36	51.66	51.84	52.01	52.04	53.06	52.95	53.49	52.60	51.73	55.55	53.16	51.85	53.18
TiO_2	1.03	1.02	1.03	1.01	1.01	0.88	0.88	0.88	1.01	1.00	1.01	0.88	0.91	0.79	0.87	0.96	1.16
Al_2O_3	17.24	17.05	17.37	15.68	15.65	13.44	13.50	13.41	15.51	15.59	15.83	16.62	16.54	15.83	15.57	14.88	14.55
Fe_2O_3	9.75	9.57	9.77	9.46	9.39	9.84	9.85	9.87	8.66	8.78	8.56	8.67	8.89	7.51	8.99	9.96	14.21
MnO	0.17	0.17	0.16	0.16	0.16	0.17	0.16	0.17	0.14	0.15	0.14	0.15	0.15	0.13	0.15	0.17	0.22
MgO	6.14	5.98	6.23	8.32	8.17	10.62	10.56	10.69	7.74	7.78	7.48	8.38	8.75	7.55	7.75	9.69	4.59
CaO	9.01	8.99	8.85	9.67	9.71	9.25	9.26	9.21	8.32	8.20	8.12	8.72	9.15	7.22	8.91	9.14	9.85
Na_2O	3.61	3.68	3.56	3.24	3.22	2.84	2.83	2.91	3.55	3.37	3.50	3.31	3.20	4.02	3.40	2.86	2.00
K_2O	1.07	1.07	1.04	1.14	1.16	1.03	1.05	1.04	1.84	1.78	1.89	0.75	0.72	1.41	1.17	0.80	0.43
P_2O_5	0.14	0.14	0.15	0.16	0.17	0.13	0.13	0.13	0.29	0.28	0.29	0.09	0.08	0.13	0.13	0.07	0.09
Total	100.30	100.54	100.56	100.21	100.30	100.03	100.23	100.35	100.12	99.88	100.31	100.15	100.10	100.13	100.10	100.37	100.27
Mg#	0.55	0.55	0.56	0.64	0.63	0.68	0.68	0.68	0.64	0.64	0.63	0.66	0.66	0.67	0.63	0.66	-
Rb (ppm)	12.51	13.66	12.12	15.60	17.92	14.47	14.48	15.30	28.33	27.39	31.54	7.62	9.71	21.74	17.67	9.54	6.28
Ba	267.31	274.28	271.14	353.99	365.20	244.20	245.39	253.76	989.28	991.25	1050.11	321.47	303.21	595.96	308.94	235.86	225.49
Th	0.52	0.52	0.51	0.59	0.59	0.61	0.62	0.62	0.83	0.88	0.91	0.83	0.80	1.19	0.62	0.47	0.28
U	0.29	0.31	0.29	0.35	0.34	0.36	0.37	0.38	0.58	0.60	0.62	0.51	0.49	0.73	0.39	0.28	0.18
Nb	1.50	1.48	1.48	1.33	1.24	1.90	1.50	1.52	1.50	1.89	1.91	4.38	4.31	1.37	1.84	1.55	0.62
Ta	0.10	0.10	0.10	0.08	0.07	0.10	0.06	0.06	0.08	0.10	0.10	0.23	0.24	0.07	0.10	0.06	0.05
La	6.27	6.34	6.03	7.01	7.10	5.87	5.92	6.14	7.84	7.93	8.15	6.53	6.42	9.03	6.28	5.36	2.32
Ce	15.60	15.88	15.51	18.03	18.35	14.69	14.83	15.41	19.94	20.36	21.09	15.65	15.42	21.79	16.08	13.98	6.43
Pb	2.28	2.41	2.32	2.39	2.26	2.23	2.25	2.21	4.64	4.54	4.67	2.94	2.85	5.97	2.76	2.04	6.80
Pr	2.52	2.54	2.48	2.81	2.86	2.31	2.33	2.45	3.14	3.17	3.28	2.27	2.28	3.16	2.50	2.21	1.11
Sr	453.60	456.02	432.47	490.74	502.31	356.95	356.79	378.15	635.07	634.45	650.36	451.78	444.01	562.99	399.70	333.37	168.76
Nd	12.95	13.03	12.59	14.24	14.54	11.72	11.89	12.56	15.84	15.93	16.44	11.09	11.15	15.10	12.69	11.30	6.39
Zr	74.09	74.23	73.55	78.51	80.45	70.44	69.45	72.36	143.96	154.37	164.80	86.77	85.90	104.29	90.41	74.65	47.20
Hf	2.01	2.01	1.99	2.13	2.13	1.94	1.95	2.00	4.07	4.24	4.56	2.19	2.18	2.76	2.41	2.00	1.61
Sm	3.29	3.33	3.24	3.59	3.66	3.01	3.06	3.23	3.89	3.94	4.03	2.87	2.93	3.55	3.30	3.05	2.35
Eu	1.13	1.14	1.10	1.19	1.21	0.98	1.00	1.06	1.32	1.32	1.37	1.03	1.04	1.14	1.09	1.04	0.87
Gd	3.57	3.57	3.44	3.88	3.98	3.28	3.33	3.61	4.18	4.17	4.30	3.40	3.51	3.86	3.63	3.56	3.16
Tb	0.52	0.52	0.51	0.57	0.58	0.50	0.51	0.54	0.59	0.59	0.60	0.52	0.54	0.55	0.55	0.56	0.59
Dy	3.16	3.15	3.06	3.46	3.51	3.09	3.14	3.35	3.45	3.51	3.51	3.31	3.42	3.30	3.43	3.55	4.15
Y	15.71	15.45	14.53	16.37	16.81	14.70	14.87	15.95	15.99	16.35	16.17	16.03	16.58	15.65	16.43	17.42	23.99
Ho	0.63	0.63	0.61	0.69	0.70	0.62	0.64	0.68	0.68	0.69	0.69	0.68	0.70	0.66	0.69	0.72	0.78
Er	1.79	1.78	1.71	1.94	1.97	1.76	1.81	1.93	1.90	1.92	1.93	1.94	2.00	1.89	1.97	2.05	2.52
Tm	0.25	0.25	0.24	0.27	0.27	0.25	0.26	0.27	0.26	0.27	0.27	0.27	0.28	0.26	0.28	0.29	0.45
Yb	1.64	1.62	1.58	1.76	1.79	1.64	1.68	1.80	1.73	1.77	1.76	1.81	1.87	1.74	1.84	1.92	2.80
Lu	0.25	0.25	0.24	0.27	0.27	0.25	0.26	0.28	0.26	0.27	0.27	0.28	0.29	0.27	0.28	0.29	0.44
Cr	81.89	85.17	83.82	269.01	271.77	484.71	458.41	499.33	235.46	240.17	223.91	319.61	360.04	249.11	282.16	442.63	21.97
Ni	42.03	39.82	38.98	70.74	70.09	125.64	121.93	130.48	110.67	112.88	106.48	119.79	128.24	175.76	72.36	144.91	12.52

Supplementary Table S3. Ni contents in each stage of the B and HMA magma genesis based on inversion modeling

		Dehydration				Mixing			Melting					Condition	Crystaliza	Raw data		Consistency
Type	Sample	C_SM[a]	D_SM[b]	s_SM[c]	C_f_SM[d]	r[e]	y[f]	C_S[g]	s[h]	t[i]	Kd_Ol[j]	D_Melt[k]	C_melt_calc	C_melt_con	Kd_Ol[n]	C_Ol[o]	C_melt_obs[p]	$C_{\_melt\_calc}/C_{\_m}$
		Ni (ppm)	$D_{Ni}^{fluid/rock}$	%	Ni (ppm)	Mantle	%	Ni (ppm)	P (GPa)	F (%)	$Kd_{Ni}^{ol/melt}$	$D_{Ni}^{rock/melt}$	Ni (ppm)	Ni (ppm)	$Kd_{Ni}^{ol/melt}$	Ni (ppm)	Ni (ppm)	log
Detroit+ Peridotite	B	99.8	4.2	2.5	389.8	2672	0.5	2658	1	14	10.3	7.4	410.8	238-282	12.2-10.3	2900	144.9	3
			2.1		200.3	1886		1876					289.9			2900		
			1.5		148.2	550		547.7					84.6			2900		
	HMA	99.8	4.2	0	421.2	2672	0.5	2661	1.5	16	10.3	7.4	419.0	466-612	13.5-10.3	6300	175.8	3.96
			2.1		205.6	1886		1878					295.7			6300		
			1.5		150.1	550		548.0					86.3			6300		
Detroit+ 17.5%Pyroxenite[r]	HMA	99.8	4.2	0.5	414.5	2601	0.4	2593	-	12	10.3	6.6	435.9	466-612	13.5-10.3	6300	175.8	4.03
			2.1		204.5	1836		1830					307.7			6300		
			1.5		149.7	536		534.0					89.8			6300		
Detroit+ 35.1%Pyroxenite[r]	HMA	99.8	4.2	0.5	414.5	2531	0.4	2523	-	11	10.3	5.9	472.5	466-612	13.5-10.3	6300	175.8	4.07
			2.1		257.7	1787		1780					333.5			6300		
			1.5		149.7	521		519.6					97.3			6300		
Detroit+ 52.6%Pyroxenite[r]	HMA	99.8	4.2	0.5	414.5	2461	0.4	2453	-	10	10.3	5.1	520.2	466-612	13.5-10.3	6300	175.8	4.11
			2.1		257.7	1737		1731					367.1			6300		
			1.5		149.7	507		505.2					107.1			6300		

[a]Typical Ni content of the Detroit Seamount.

[b]Fluid–rock partition coefficient of Ni from ref. 71.

[c]Amount of dehydration from the Detroit Seamount.

[d]Ni content of slab-derived fluid.

[e]Ni content of original peridotite and/or pyroxenite based on DMM[72] and the Kamchatka peridotite mantle xenolith[34]. Assuming ~30% forsteritic olivine in peridotite, ~8% of silica is required to transform olivine to orthopyroxene (~52.6% pyroxenite)[23].

[f]Mixing ratio of slab-derived fluid and original mantle.

[g]Ni content of source mantle.

[h]Melting pressure.

[i]Degree of melting.

[j]Ol/melt partition coefficient of Ni ($Kd_{Ni}^{ol/melt}$) for partial melting of mantle peridotite after ref. 81.

[k]Bulk partition coefficient of Ni ($D_{Ni}^{ol/melt}$) after ref. 81.

[l]Calculated Ni content of melt.

[m]Condition of Ni content in melt to generate maximum Ni value of Ol based on $Kd_{Ni}^{Ol/melt}$ at crystallization.

[n]Ol/melt partition coefficient of Ni ($Kd_{Ni}^{ol/melt}$) for crystallization from primitive melt after refs. 81, 82.

[o]Maximum Ni content of Ol measured by EPMA.

[p]Ni content of bulk rock measured by ICP-MS.

[q]Residual between composition of the calculation and observation value. $\Sigma \log_{10}$(C_melt_calc/C_melt_obs) for all 27 elements.

[r]Transformation ratio of mantle olivine to orthopyroxene in 10 vol.% steps by Si-rich slab fluid.

Italic type: free parameter, **bold type**: Ni content in each calculation step.

Supplementary Table S4. **Isotopic compositions of $^{87}Sr/^{86}Sr$, $^{143}Nd/^{144}Nd$, $^{206}Pb/^{204}Pb$, $^{207}Pb/^{204}Pb$, and $^{208}Pb/^{204}Pb$ in the representative samples of the EC lavas.**

Sample	$^{87}Sr/^{86}Sr$	2SE	$^{143}Nd/^{144}Nd$	2SE	$^{206}Pb/^{204}Pb$	2SE	$^{207}Pb/^{204}Pb$	2SE	$^{208}Pb/^{204}Pb$	2SE
EC-13-1/1	0.703428	0.000006	0.513110	0.000009	18.2600	0.0011	15.4775	0.0010	37.8389	0.0026
EC-13-1/2	0.703422	0.000006	0.513111	0.000007	18.2404	0.0008	15.4726	0.0008	37.8041	0.0020
EC-13-1/3	0.703430	0.000006	0.513108	0.000008	18.2404	0.0008	15.4724	0.0007	37.8022	0.0018
EC-13-2/1	0.703501	0.000006	0.513090	0.000007	18.2448	0.0012	15.4760	0.0011	37.8439	0.0027
EC-13-2/4	0.703481	0.000007	0.513086	0.000007	18.2443	0.0008	15.4763	0.0007	37.8432	0.0019
EC-13-3/1	0.703486	0.000006	0.513111	0.000008	18.2606	0.0019	15.4781	0.0015	37.8405	0.0037
EC-13-3/2	0.703492	0.000006	0.513106	0.000009	18.2582	0.0018	15.4769	0.0015	37.8353	0.0037
EC-13-3/4	0.703492	0.000006	0.513100	0.000007	18.2584	0.0020	15.4781	0.0016	37.8381	0.0042
EC-13-4/1	0.703624	0.000006	0.513085	0.000010	18.2982	0.0018	15.4834	0.0015	37.8728	0.0037
EC-13-4/2	0.703616	0.000006	0.513091	0.000007	18.3004	0.0008	15.4836	0.0007	37.8762	0.0018
EC-13-4/3	0.703608	0.000006	0.513088	0.000009	18.3031	0.0009	15.4842	0.0008	37.8790	0.0020
EC-13-5/1	0.703299	0.000007	0.513073	0.000008	18.2497	0.0016	15.4796	0.0013	37.8623	0.0032
EC-13-5/2	0.703297	0.000005	0.513066	0.000007	18.2469	0.0013	15.4804	0.0011	37.8626	0.0027
EC-13-6/1	0.703518	0.000005	0.513073	0.000007	18.2882	0.0012	15.4880	0.0010	37.9119	0.0027
EC-13-7/1	0.703543	0.000006	0.513109	0.000007	18.2368	0.0015	15.4733	0.0012	37.7934	0.0031
EC-13-8/1	0.703498	0.000006	0.513099	0.000008	18.2555	0.0013	15.4788	0.0010	37.8283	0.0027

Supplementary Table S5. Olivine compositions.

Sample	EC-13-6/1											
Rock-type	HMA											
Ol-type	Type-1											
Analysis po	EC-13-6/1_	EC-13-6/1_	EC-13-6/1_	EC-13-6/1_	EC-13-6/1_	EC-13-6/1_	EC-13-6/1_	EC-13-6/1_	EC-13-6/1_	EC-13-6/1_	EC-13-6/1_	EC-13-6/1_
SiO_2 (wt.%	39.29	39.95	40.66	40.15	40.55	40.73	40.71	40.44	40.81	40.68	40.98	40.59
TiO_2	0.001	0.008	0	0	0	0	0	0	0	0	0.003	0
NiO	0.45	0.58	0.66	0.71	0.74	0.75	0.75	0.75	0.81	0.75	0.73	0.72
Al_2O_3	0.01	0.02	0.02	0.00	0.02	0.02	0.00	0.01	0.01	0.02	0.03	0.00
CaO	0.09	0.10	0.11	0.08	0.09	0.09	0.10	0.08	0.07	0.08	0.10	0.08
FeO*	13.99	11.87	10.60	10.32	9.94	9.88	9.93	9.89	9.77	9.91	9.98	10.10
MgO	46.51	47.44	48.29	48.24	48.30	48.28	48.16	48.41	48.37	48.21	48.18	48.10
K_2O	0.00	0.01	0.00	0.00	0.02	0.00	0.00	0.00	0.00	0.00	0.00	0.00
MnO	0.25	0.17	0.15	0.15	0.13	0.16	0.16	0.11	0.16	0.10	0.12	0.13
Na_2O	0.02	0.01	0.00	0.00	0.00	0.01	0.00	0.02	0.02	0.00	0.00	0.00
Cr_2O_3	0.00	0.01	0.00	0.00	0.02	0.00	0.01	0.00	0.02	0.01	0.01	0.03
Total	100.61	100.16	100.47	99.64	99.79	99.92	99.82	99.70	100.05	99.76	100.11	99.74
Fo	85.6	87.7	89.0	89.3	89.6	89.7	89.6	89.7	89.8	89.7	89.6	89.5

Supplementary Table S5. (continued)

					Type-2							
EC-13-6/1_	EC-13-6/1_	EC-13-6/1_	EC-13-6/1_	EC-13-6/1_	EC-13-6/1_	EC-13-6/1_	EC-13-6/1_	EC-13-6/1_	EC-13-6/1_	EC-13-6/1_	EC-13-6/1_	EC-13-6/1_
40.64	40.56	40.10	39.94	39.13	40.11	40.57	40.19	40.50	39.60	40.70	40.30	40.74
0	0.013	0	0	0.027	0.016	0	0.011	0	0	0	0	0
0.64	0.57	0.45	0.32	0.17	0.49	0.53	0.53	0.58	0.61	0.60	0.64	0.62
0.00	0.00	0.00	0.04	0.02	0.01	0.00	0.01	0.02	0.00	0.01	0.00	0.00
0.09	0.05	0.10	0.09	0.10	0.09	0.10	0.10	0.06	0.08	0.08	0.09	0.09
10.72	10.95	11.56	13.08	16.58	11.62	11.20	11.09	10.82	10.45	10.50	10.50	10.24
47.53	47.24	46.81	45.41	41.45	47.65	47.58	47.58	47.79	47.97	48.05	48.05	47.90
0.01	0.01	0.00	0.01	0.00	0.00	0.00	0.00	0.00	0.00	0.00	0.00	0.01
0.14	0.18	0.16	0.22	0.31	0.17	0.16	0.19	0.13	0.12	0.12	0.14	0.16
0.00	0.01	0.00	0.00	0.00	0.04	0.00	0.00	0.02	0.00	0.00	0.00	0.01
0.04	0.00	0.01	0.01	0.03	0.04	0.00	0.00	0.01	0.00	0.02	0.07	0.07
99.81	99.57	99.19	99.11	97.81	100.23	100.14	99.71	99.92	98.82	100.08	99.79	99.83
88.8	88.5	87.8	86.1	81.7	88.0	88.3	88.4	88.7	89.1	89.1	89.1	89.3

Supplementary Table S5. (continued)

												Type-3
EC-13-6/1_	EC-13-6/1_	EC-13-6/1_	EC-13-6/1_	EC-13-6/1_	EC-13-6/1_	EC-13-6/1_	EC-13-6/1_	EC-13-6/1_	EC-13-6/1_	EC-13-6/1_	EC-13-6/1_	EC-13-6/1_
40.88	40.54	40.32	40.71	40.65	40.70	40.73	40.59	40.52	40.64	40.33	40.82	40.43
0.007	0	0	0	0.019	0	0	0.016	0	0	0.004	0.047	0
0.62	0.61	0.59	0.60	0.58	0.56	0.61	0.55	0.56	0.56	0.57	0.57	0.47
0.01	0.00	0.00	0.01	0.00	0.04	0.00	0.00	0.00	0.04	0.00	0.02	0.00
0.08	0.05	0.10	0.09	0.07	0.09	0.08	0.08	0.07	0.07	0.08	0.10	0.06
10.15	10.19	10.15	10.05	10.01	10.05	10.09	10.09	9.95	10.18	10.10	10.14	10.01
48.32	48.13	48.34	47.99	48.12	48.12	48.05	48.10	47.80	47.89	47.47	46.60	49.55
0.01	0.01	0.01	0.00	0.01	0.00	0.01	0.01	0.01	0.00	0.00	0.00	0.01
0.17	0.12	0.11	0.14	0.14	0.14	0.10	0.14	0.11	0.11	0.16	0.13	0.16
0.03	0.00	0.00	0.01	0.02	0.00	0.03	0.03	0.00	0.00	0.02	0.00	0.00
0.00	0.01	0.03	0.00	0.00	0.04	0.01	0.00	0.03	0.03	0.04	0.03	0.01
100.27	99.67	99.64	99.60	99.62	99.74	99.71	99.59	99.05	99.50	98.76	98.45	100.69
89.5	89.4	89.5	89.5	89.5	89.5	89.5	89.5	89.5	89.3	89.3	89.1	89.8

Supplementary Table S5. (continued)

EC-13-6/1_	EC-13-6/1_	EC-13-6/1_	EC-13-6/1_	EC-13-6/1_	EC-13-6/1_	EC-13-6/1_	EC-13-6/1_	EC-13-6/1_	EC-13-6/1_	EC-13-6/1_	EC-13-6/1_	EC-13-6/1_
40.76	40.67	40.82	40.67	40.78	40.70	40.68	40.56	40.51	41.01	40.80	40.99	40.56
0.028	0.007	0.007	0	0.036	0.045	0	0	0.024	0	0.001	0.001	0
0.50	0.51	0.51	0.49	0.50	0.49	0.46	0.49	0.51	0.47	0.46	0.49	0.49
0.01	0.00	0.01	0.00	0.00	0.02	0.04	0.00	0.00	0.01	0.00	0.01	0.03
0.07	0.08	0.10	0.09	0.07	0.09	0.10	0.09	0.09	0.09	0.09	0.09	0.09
10.29	10.16	10.06	10.01	10.25	10.17	9.92	10.03	10.11	10.13	10.37	9.88	10.29
48.92	48.40	48.38	48.03	48.29	48.24	48.35	48.11	48.48	48.35	48.38	48.03	48.13
0.00	0.01	0.01	0.00	0.01	0.00	0.00	0.00	0.00	0.01	0.01	0.01	0.00
0.13	0.15	0.17	0.13	0.17	0.12	0.12	0.13	0.11	0.15	0.18	0.17	0.14
0.02	0.02	0.00	0.00	0.00	0.02	0.01	0.04	0.00	0.02	0.00	0.00	0.00
0.00	0.03	0.03	0.00	0.00	0.00	0.01	0.00	0.02	0.06	0.04	0.00	0.00
100.72	100.04	100.11	99.43	100.10	99.91	99.69	99.46	99.84	100.29	100.33	99.65	99.73
89.4	89.5	89.6	89.5	89.4	89.4	89.7	89.5	89.5	89.5	89.3	89.7	89.3

Supplementary Table S5. (continued)

						Type-1						
EC-13-6/1_	EC-13-6/1_	EC-13-6/1_	EC-13-6/1_	EC-13-6/1_	EC-13-6/1_	EC-13-6/1_	EC-13-6/1_	EC-13-6/1_	EC-13-6/1_	EC-13-6/1_	EC-13-6/1_	EC-13-6/1_
40.66	40.68	40.81	40.70	40.55	41.48	40.58	40.19	40.37	40.31	40.38	40.08	40.17
0	0.028	0	0.032	0	0	0	0	0.022	0.014	0.001	0	0
0.48	0.49	0.48	0.50	0.48	0.50	0.15	0.20	0.23	0.32	0.25	0.35	0.32
0.03	0.01	0.00	0.02	0.01	0.01	0.00	0.00	0.00	0.01	0.03	0.00	0.03
0.08	0.08	0.08	0.11	0.10	0.10	0.13	0.12	0.10	0.09	0.12	0.08	0.09
10.11	10.11	10.12	10.17	10.10	9.52	13.69	12.85	12.34	11.83	11.93	11.63	11.51
48.03	48.22	48.04	47.78	47.10	49.09	45.04	45.99	45.99	46.68	46.56	47.21	47.17
0.00	0.01	0.01	0.00	0.00	0.02	0.00	0.00	0.00	0.00	0.00	0.00	0.00
0.12	0.08	0.17	0.12	0.12	0.13	0.22	0.15	0.18	0.16	0.19	0.17	0.19
0.03	0.02	0.00	0.02	0.00	0.04	0.00	0.02	0.00	0.00	0.00	0.00	0.00
0.00	0.00	0.00	0.01	0.00	0.13	0.00	0.00	0.00	0.01	0.00	0.00	0.00
99.54	99.70	99.71	99.47	98.46	101.01	99.81	99.53	99.22	99.43	99.46	99.51	99.48
89.4	89.5	89.4	89.3	89.3	90.2	85.4	86.4	86.9	87.5	87.4	87.9	88.0

Supplementary Table S5. (continued)

				Type-2								
EC-13-6/1_	EC-13-6/1_	EC-13-6/1_	EC-13-6/1_	EC-13-6/1_	EC-13-6/1_	EC-13-6/1_	EC-13-6/1_	EC-13-6/1_	EC-13-6/1_	EC-13-6/1_	EC-13-6/1_	EC-13-6/1_
40.24	39.73	38.81	40.12	39.74	40.39	40.00	40.11	40.43	40.27	40.37	40.53	40.39
0	0	0.03	0	0	0.001	0	0.002	0	0	0	0	0
0.22	0.17	0.12	0.33	0.17	0.21	0.29	0.37	0.42	0.41	0.36	0.35	0.42
0.00	0.03	0.00	0.00	0.00	0.00	0.00	0.03	0.00	0.00	0.01	0.00	0.01
0.09	0.10	0.10	0.09	0.13	0.09	0.08	0.07	0.09	0.09	0.09	0.11	0.09
12.15	13.04	14.51	11.40	15.29	14.00	13.06	12.26	12.01	11.74	11.55	11.24	11.32
46.67	45.88	43.98	47.11	44.26	45.43	45.91	46.28	46.77	46.78	47.19	47.38	47.29
0.00	0.01	0.00	0.00	0.00	0.00	0.00	0.00	0.00	0.00	0.00	0.00	0.00
0.17	0.21	0.19	0.17	0.29	0.20	0.21	0.15	0.15	0.19	0.18	0.14	0.14
0.00	0.00	0.00	0.02	0.00	0.00	0.00	0.00	0.01	0.01	0.03	0.00	0.00
0.00	0.00	0.00	0.05	0.00	0.03	0.05	0.00	0.00	0.00	0.02	0.00	0.07
99.56	99.16	97.75	99.29	99.87	100.34	99.59	99.28	99.89	99.49	99.80	99.75	99.72
87.3	86.2	84.4	88.0	83.8	85.3	86.2	87.1	87.4	87.7	87.9	88.3	88.2

Supplementary Table S5. (continued)

		Type-1										
EC-13-6/1_	EC-13-6/1_	EC-13-6/1_	EC-13-6/1_	EC-13-6/1_	EC-13-6/1_	EC-13-6/1_	EC-13-6/1_	EC-13-6/1_	EC-13-6/1_	EC-13-6/1_	EC-13-6/1_	EC-13-6/1_
39.87	40.39	40.59	40.26	40.13	40.44	40.62	40.11	39.93	39.97	40.13	39.29	40.21
0.024	0	0.005	0	0	0.002	0	0	0.026	0	0	0.042	0
0.45	0.44	0.39	0.38	0.42	0.45	0.42	0.43	0.38	0.37	0.38	0.32	0.45
0.03	0.01	0.02	0.00	0.02	0.00	0.01	0.03	0.02	0.00	0.03	0.00	0.04
0.09	0.09	0.10	0.09	0.09	0.08	0.09	0.09	0.10	0.08	0.09	0.09	0.10
11.54	11.44	11.23	11.06	10.72	10.52	10.81	11.05	11.72	12.22	11.84	13.41	10.95
46.50	47.62	47.99	47.94	47.64	47.91	47.87	47.40	46.70	46.39	46.75	43.05	48.44
0.01	0.00	0.00	0.00	0.01	0.00	0.00	0.00	0.01	0.01	0.01	0.00	0.00
0.19	0.21	0.17	0.15	0.17	0.17	0.13	0.16	0.20	0.18	0.16	0.21	0.13
0.00	0.00	0.01	0.00	0.02	0.01	0.01	0.01	0.00	0.01	0.01	0.00	0.01
0.02	0.09	0.03	0.01	0.00	0.00	0.04	0.00	0.00	0.00	0.00	0.00	0.07
98.72	100.28	100.52	99.89	99.23	99.58	99.99	99.28	99.08	99.23	99.39	96.41	100.38
87.8	88.1	88.4	88.5	88.8	89.0	88.8	88.4	87.7	87.1	87.6	85.1	88.7

Supplementary Table S5. (continued)

Type-1				Type-2							Type-3	
EC-13-6/1_	EC-13-6/1_	EC-13-6/1_	EC-13-6/1_	EC-13-6/1_	EC-13-6/1_	EC-13-6/1_	EC-13-6/1_	EC-13-6/1_	EC-13-6/1_	EC-13-6/1_	EC-13-6/1_	EC-13-6/1_
39.73	39.75	39.16	39.70	40.47	40.57	40.59	40.32	40.85	40.17	40.55	39.23	40.82
0	0.035	0	0	0	0.003	0.036	0	0	0	0	0.014	0
0.36	0.26	0.19	0.18	0.39	0.42	0.39	0.44	0.40	0.38	0.42	0.18	0.51
0.02	0.01	0.00	0.00	0.00	0.00	0.00	0.04	0.03	0.02	0.02	0.01	0.00
0.08	0.10	0.12	0.07	0.08	0.06	0.11	0.07	0.11	0.10	0.10	0.10	0.09
12.22	14.25	17.82	16.27	10.59	10.17	10.14	10.77	10.24	10.14	10.47	17.53	10.49
47.99	44.64	39.78	41.78	48.88	48.29	47.92	46.28	48.26	48.69	48.10	43.47	48.15
0.00	0.00	0.01	0.00	0.00	0.00	0.00	0.00	0.00	0.01	0.00	0.00	0.01
0.16	0.21	0.36	0.27	0.13	0.17	0.11	0.18	0.12	0.14	0.14	0.31	0.11
0.00	0.00	0.00	0.00	0.00	0.00	0.00	0.00	0.01	0.03	0.01	0.00	0.00
0.01	0.00	0.00	0.05	0.00	0.00	0.00	0.00	0.16	0.15	0.08	0.00	0.01
100.57	99.25	97.43	98.33	100.55	99.68	99.31	98.10	100.17	99.83	99.89	100.84	100.19
87.5	84.8	79.9	82.1	89.2	89.4	89.4	88.5	89.4	89.5	89.1	81.6	89.1

Supplementary Table S5. (continued)

					-			Type-1				Type-2
EC-13-6/1_	EC-13-6/1_	EC-13-6/1_	EC-13-6/1_	EC-13-6/1_	EC-13-6/1_	EC-13-6/1_	EC-13-6/1_	EC-13-6/1_	EC-13-6/1_	EC-13-6/1_	EC-13-6/1_	EC-13-6/1_
40.59	41.20	40.72	40.63	41.06	38.56	41.11	40.47	38.77	40.29	40.80	40.38	38.50
0.006	0.019	0	0	0	0.018	0	0.016	0.016	0	0	0	0.01
0.36	0.45	0.44	0.50	0.49	0.11	0.49	0.11	0.24	0.49	0.42	0.54	0.26
0.04	0.04	0.03	0.02	0.05	0.01	0.02	0.00	0.01	0.00	0.01	0.03	0.01
0.09	0.10	0.12	0.10	0.10	0.10	0.08	0.11	0.12	0.09	0.10	0.11	0.12
10.17	10.51	11.95	10.11	10.50	16.78	9.84	17.14	15.62	10.72	12.58	10.66	14.65
48.88	48.85	46.58	48.75	47.64	44.55	47.86	39.84	46.03	47.72	44.62	47.75	46.03
0.01	0.00	0.02	0.01	0.00	0.00	0.01	0.02	0.00	0.00	0.00	0.02	0.00
0.11	0.15	0.21	0.12	0.14	0.30	0.12	0.31	0.31	0.13	0.18	0.12	0.28
0.04	0.00	0.00	0.00	0.00	0.00	0.00	0.01	0.00	0.00	0.00	0.00	0.00
0.00	0.00	0.00	0.11	0.27	0.00	0.05	0.00	0.00	0.00	0.00	0.02	0.00
100.29	101.32	100.08	100.34	100.25	100.42	99.58	98.03	101.13	99.45	98.71	99.62	99.85
89.5	89.2	87.4	89.6	89.0	82.6	89.7	80.6	84.0	88.8	86.3	88.9	84.9

Supplementary Table S5. (continued)

						Type-1			Type-1			-
EC-13-6/1_	EC-13-6/1_	EC-13-6/1_	EC-13-6/1_	EC-13-6/1_	EC-13-6/1_	EC-13-6/1_	EC-13-6/1_	EC-13-6/1_	EC-13-6/1_	EC-13-6/1_	EC-13-6/1_	EC-13-6/1_
40.55	40.60	40.95	40.61	40.51	40.19	39.49	40.46	40.23	39.05	40.54	39.77	40.01
0	0.015	0	0	0.01	0	0.016	0	0.053	0.025	0	0	0.029
0.62	0.48	0.55	0.21	0.52	0.59	0.19	0.50	0.25	0.24	0.28	0.22	0.50
0.00	0.00	0.00	0.02	0.02	0.00	0.03	0.02	0.00	0.01	0.00	0.03	0.00
0.08	0.10	0.07	0.10	0.13	0.09	0.10	0.06	0.08	0.10	0.12	0.07	0.09
10.25	10.00	10.13	14.52	9.76	10.59	15.83	10.33	13.98	15.10	11.22	16.18	10.80
48.22	48.62	47.96	42.33	48.30	47.98	44.66	48.34	43.57	46.22	47.51	41.74	49.14
0.00	0.01	0.01	0.00	0.00	0.00	0.00	0.01	0.02	0.00	0.00	0.02	0.00
0.11	0.14	0.13	0.28	0.11	0.16	0.29	0.17	0.22	0.27	0.19	0.29	0.15
0.00	0.02	0.00	0.00	0.01	0.01	0.01	0.00	0.00	0.00	0.00	0.00	0.00
0.00	0.00	0.00	0.00	0.27	0.00	0.00	0.00	0.00	0.00	0.00	0.00	0.00
99.84	99.97	99.81	98.07	99.64	99.60	100.61	99.88	98.40	101.01	99.86	98.31	100.72
89.3	89.7	89.4	83.9	89.8	89.0	83.4	89.3	84.7	84.5	88.3	82.1	89.0

Supplementary Table S5. (continued)

	Type-1						-		Type-2			
EC-13-6/1_	EC-13-6/1_	EC-13-6/1_	EC-13-6/1_	EC-13-6/1_	EC-13-6/1_	EC-13-6/1_	EC-13-6/1_	EC-13-6/1_	EC-13-6/1_	EC-13-6/1_	EC-13-6/1_	EC-13-6/1_
40.40	39.87	40.55	40.95	40.50	40.13	40.42	39.57	40.11	39.94	40.26	40.20	40.10
0	0.01	0	0.03	0.016	0.001	0	0	0.023	0	0	0	0
0.45	0.16	0.44	0.44	0.38	0.24	0.47	0.29	0.45	0.66	0.68	0.62	0.64
0.01	0.01	0.02	0.00	0.03	0.03	0.00	0.01	0.03	0.02	0.01	0.00	0.00
0.09	0.11	0.10	0.07	0.07	0.09	0.07	0.09	0.09	0.10	0.09	0.07	0.10
10.50	15.50	10.74	11.54	12.57	14.25	10.57	14.29	10.98	11.13	11.06	11.57	12.25
48.04	45.63	48.01	47.70	46.26	43.85	48.57	46.83	47.50	49.06	47.48	46.89	46.33
0.00	0.00	0.00	0.00	0.00	0.00	0.00	0.00	0.00	0.00	0.01	0.00	0.00
0.14	0.24	0.15	0.16	0.20	0.22	0.11	0.19	0.17	0.17	0.19	0.15	0.15
0.00	0.00	0.00	0.00	0.00	0.00	0.00	0.02	0.00	0.00	0.04	0.02	0.01
0.00	0.00	0.00	0.00	0.00	0.00	0.04	0.00	0.00	0.00	0.00	0.00	0.00
99.62	101.53	100.02	100.89	100.03	98.80	100.25	101.30	99.34	101.07	99.81	99.52	99.58
89.1	84.0	88.9	88.0	86.8	84.6	89.1	85.4	88.5	88.7	88.4	87.8	87.1

Supplementary Table S5. (continued)

	Type-1						Type-1					Type-2
EC-13-6/1_	EC-13-6/1_	EC-13-6/1_	EC-13-6/1_	EC-13-6/1_	EC-13-6/1_	EC-13-6/1_	EC-13-6/1_	EC-13-6/1_	EC-13-6/1_	EC-13-6/1_	EC-13-6/1_	EC-13-6/1_
40.33	38.85	40.86	39.99	40.79	39.61	40.75	38.84	40.44	40.58	40.41	40.09	39.46
0	0.016	0	0.014	0	0	0	0	0	0	0	0	0
0.66	0.10	0.33	0.48	0.38	0.18	0.43	0.17	0.60	0.64	0.58	0.12	0.33
0.02	0.02	0.01	0.01	0.00	0.03	0.01	0.03	0.02	0.02	0.02	0.00	0.00
0.08	0.10	0.08	0.08	0.07	0.12	0.08	0.12	0.09	0.09	0.07	0.11	0.09
11.13	17.28	11.81	10.28	10.69	16.08	10.49	16.11	10.66	10.31	11.03	18.09	13.45
45.97	44.07	46.64	49.68	48.16	42.74	47.99	45.54	48.00	48.66	47.89	39.94	47.02
0.00	0.00	0.01	0.00	0.00	0.00	0.01	0.01	0.00	0.00	0.00	0.00	0.00
0.19	0.31	0.11	0.13	0.13	0.28	0.11	0.35	0.16	0.13	0.15	0.35	0.21
0.02	0.01	0.00	0.00	0.02	0.01	0.00	0.03	0.03	0.00	0.00	0.00	0.00
0.00	0.00	0.00	0.07	0.00	0.00	0.00	0.00	0.00	0.00	0.00	0.00	0.00
98.41	100.75	99.86	100.73	100.23	99.04	99.87	101.19	99.99	100.43	100.15	98.71	100.55
88.0	82.0	87.6	89.6	88.9	82.6	89.1	83.4	88.9	89.4	88.6	79.7	86.2

Supplementary Table S5. (continued)

	Type-1						Type-1					Type-2
EC-13-6/1_	EC-13-6/1_	EC-13-6/1_	EC-13-6/1_	EC-13-6/1_	EC-13-6/1_	EC-13-6/1_	EC-13-6/1_	EC-13-6/1_	EC-13-6/1_	EC-13-6/1_	EC-13-6/1_	EC-13-6/1_
40.33	38.85	40.86	39.99	40.79	39.61	40.75	38.84	40.44	40.58	40.41	40.09	39.46
0	0.016	0	0.014	0	0	0	0	0	0	0	0	0
0.66	0.10	0.33	0.48	0.38	0.18	0.43	0.17	0.60	0.64	0.58	0.12	0.33
0.02	0.02	0.01	0.01	0.00	0.03	0.01	0.03	0.02	0.02	0.02	0.00	0.00
0.08	0.10	0.08	0.08	0.07	0.12	0.08	0.12	0.09	0.09	0.07	0.11	0.09
11.13	17.28	11.81	10.28	10.69	16.08	10.49	16.11	10.66	10.31	11.03	18.09	13.45
45.97	44.07	46.64	49.68	48.16	42.74	47.99	45.54	48.00	48.66	47.89	39.94	47.02
0.00	0.00	0.01	0.00	0.00	0.00	0.01	0.01	0.00	0.00	0.00	0.00	0.00
0.19	0.31	0.11	0.13	0.13	0.28	0.11	0.35	0.16	0.13	0.15	0.35	0.21
0.02	0.01	0.00	0.00	0.02	0.01	0.00	0.03	0.03	0.00	0.00	0.00	0.00
0.00	0.00	0.00	0.07	0.00	0.00	0.00	0.00	0.00	0.00	0.00	0.00	0.00
98.41	100.75	99.86	100.73	100.23	99.04	99.87	101.19	99.99	100.43	100.15	98.71	100.55
88.0	82.0	87.6	89.6	88.9	82.6	89.1	83.4	88.9	89.4	88.6	79.7	86.2

Supplementary Table S5. (continued)

					-		-			Type-2		Type-1
EC-13-6/1_	EC-13-6/1_	EC-13-6/1_	EC-13-6/1_	EC-13-6/1_	EC-13-6/1_	EC-13-6/1_	EC-13-6/1_	EC-13-6/1_	EC-13-6/1_	EC-13-6/1_	EC-13-6/1_	EC-13-6/1_
40.52	40.78	40.48	41.21	40.84	39.35	40.93	39.41	40.47	40.51	40.31	40.78	39.35
0	0	0.009	0.029	0.012	0	0	0	0	0	0	0	0
0.64	0.64	0.58	0.46	0.65	0.57	0.67	0.31	0.42	0.47	0.50	0.46	0.20
0.03	0.00	0.00	0.00	0.01	0.00	0.00	0.01	0.00	0.00	0.02	0.00	0.02
0.09	0.06	0.06	0.07	0.06	0.10	0.08	0.12	0.08	0.12	0.10	0.09	0.09
10.37	10.33	11.07	11.85	10.71	10.93	11.15	14.01	10.75	10.76	13.90	10.66	14.50
48.25	48.27	47.49	45.08	48.00	50.49	47.54	46.78	48.23	48.10	46.96	48.54	46.64
0.00	0.00	0.02	0.00	0.00	0.00	0.00	0.02	0.00	0.00	0.00	0.00	0.00
0.11	0.13	0.15	0.18	0.16	0.14	0.15	0.21	0.12	0.16	0.23	0.14	0.25
0.02	0.04	0.02	0.02	0.01	0.00	0.00	0.02	0.00	0.01	0.00	0.00	0.00
0.00	0.00	0.00	0.05	0.00	0.00	0.00	0.00	0.00	0.08	0.00	0.09	0.00
100.01	100.25	99.90	98.94	100.45	101.57	100.52	100.89	100.06	100.22	102.00	100.78	101.05
89.2	89.3	88.4	87.2	88.9	89.2	88.4	85.6	88.9	88.8	85.8	89.0	85.1

Supplementary Table S5. (continued)

		Type-1			Type-2			Type-2				
EC-13-6/1_	EC-13-6/1_	EC-13-6/1_	EC-13-6/1_	EC-13-6/1_	EC-13-6/1_	EC-13-6/1_	EC-13-6/1_	EC-13-6/1_	EC-13-6/1_	EC-13-6/1_	EC-13-6/1_	EC-13-6/1_
40.39	40.53	39.64	40.71	40.54	38.47	40.87	40.71	38.45	40.59	41.13	40.87	41.07
0	0.019	0	0.005	0	0.026	0	0	0.03	0.021	0.026	0.008	0
0.44	0.31	0.37	0.38	0.36	0.29	0.51	0.65	0.13	0.47	0.55	0.42	0.40
0.00	0.00	0.00	0.00	0.00	0.00	0.00	0.00	0.00	0.00	0.02	0.00	0.02
0.08	0.08	0.09	0.12	0.11	0.06	0.07	0.06	0.12	0.08	0.11	0.09	0.09
10.96	14.24	12.85	11.25	12.46	16.87	9.92	10.33	17.72	11.25	10.70	10.56	10.67
47.69	42.76	48.16	47.58	44.46	44.44	49.02	46.52	44.59	47.83	48.06	48.30	48.15
0.00	0.00	0.01	0.01	0.00	0.00	0.00	0.01	0.00	0.00	0.01	0.00	0.00
0.17	0.24	0.15	0.16	0.18	0.32	0.12	0.11	0.32	0.19	0.15	0.16	0.15
0.02	0.00	0.02	0.00	0.00	0.00	0.00	0.00	0.00	0.00	0.00	0.03	0.00
0.02	0.08	0.00	0.00	0.22	0.00	0.03	0.04	0.00	0.02	0.00	0.00	0.00
99.76	98.26	101.30	100.23	98.34	100.47	100.55	98.43	101.35	100.45	100.74	100.43	100.53
88.6	84.3	87.0	88.3	86.4	82.4	89.8	88.9	81.8	88.3	88.9	89.1	88.9

Supplementary Table S5. (continued)

						Type-1				Type-1		
EC-13-6/1_	EC-13-6/1_	EC-13-6/1_	EC-13-6/1_	EC-13-6/1_	EC-13-6/1_	EC-13-6/1_	EC-13-6/1_	EC-13-6/1_	EC-13-6/1_	EC-13-6/1_	EC-13-6/1_	EC-13-6/1_
40.72	40.79	40.72	40.96	41.59	40.61	38.99	40.57	40.13	40.61	39.17	40.47	40.57
0	0	0.027	0	0.037	0.04	0	0	0	0	0	0	0
0.35	0.34	0.41	0.40	0.50	0.39	0.27	0.57	0.31	0.60	0.48	0.42	0.41
0.00	0.01	0.01	0.01	0.00	0.04	0.00	0.01	0.00	0.00	0.02	0.01	0.02
0.10	0.09	0.15	0.08	0.08	0.15	0.09	0.08	0.15	0.09	0.09	0.10	0.08
10.92	10.63	10.66	10.65	11.42	10.77	16.98	12.23	15.81	11.47	10.55	10.70	10.70
48.46	48.49	47.96	47.94	44.78	47.97	44.39	46.91	41.53	47.53	50.27	48.53	48.46
0.00	0.00	0.02	0.00	0.01	0.01	0.00	0.01	0.00	0.00	0.00	0.01	0.02
0.16	0.16	0.16	0.13	0.18	0.16	0.28	0.19	0.32	0.16	0.15	0.15	0.15
0.00	0.00	0.00	0.00	0.00	0.00	0.00	0.00	0.01	0.00	0.02	0.00	0.02
0.00	0.03	0.03	0.00	0.00	0.00	0.00	0.00	0.04	0.00	0.00	0.00	0.00
100.71	100.55	100.15	100.17	98.59	100.14	100.99	100.57	98.30	100.48	100.74	100.39	100.44
88.8	89.0	88.9	88.9	87.5	88.8	82.3	87.2	82.4	88.1	89.5	89.0	89.0

Supplementary Table S5. (continued)

			Type-1			Type-2					Type-1	
EC-13-6/1_	EC-13-6/1_	EC-13-6/1_	EC-13-6/1_	EC-13-6/1_	EC-13-6/1_	EC-13-6/1_	EC-13-6/1_	EC-13-6/1_	EC-13-6/1_	EC-13-6/1_	EC-13-6/1_	EC-13-6/1_
40.95	41.70	41.00	39.37	40.36	40.50	40.01	41.01	40.22	40.54	41.78	38.39	39.61
0.002	0.006	0.01	0	0	0	0	0	0	0.01	0.01	0	0.01
0.41	0.43	0.45	0.23	0.48	0.54	0.55	0.61	0.51	0.54	0.53	0.12	0.13
0.00	0.03	0.00	0.03	0.00	0.04	0.04	0.00	0.02	0.01	0.00	0.00	0.03
0.08	0.08	0.07	0.09	0.09	0.09	0.08	0.09	0.07	0.10	0.08	0.10	0.10
10.62	10.47	10.59	15.31	12.33	11.26	10.99	10.58	10.77	10.78	10.49	17.07	16.29
48.36	45.50	47.61	45.79	47.33	45.49	49.96	47.15	48.62	48.55	45.53	44.74	43.59
0.01	0.00	0.00	0.00	0.00	0.01	0.02	0.01	0.00	0.00	0.00	0.00	0.00
0.17	0.14	0.16	0.28	0.20	0.18	0.18	0.14	0.14	0.12	0.18	0.33	0.26
0.00	0.02	0.00	0.01	0.00	0.01	0.00	0.04	0.02	0.00	0.00	0.00	0.01
0.00	0.17	0.15	0.00	0.00	0.18	0.00	0.13	0.00	0.00	0.13	0.00	0.05
100.60	98.53	100.04	101.11	100.79	98.29	101.83	99.74	100.38	100.64	98.74	100.75	100.09
89.0	88.6	88.9	84.2	87.3	87.8	89.0	88.8	88.9	88.9	88.6	82.4	82.7

Supplementary Table S5. (continued)

	-			-			Type-1					
EC-13-6/1_	EC-13-6/1_	EC-13-6/1_	EC-13-6/1_	EC-13-6/1_	EC-13-6/1_	EC-13-6/1_	EC-13-6/1_	EC-13-6/1_	EC-13-6/1_	EC-13-6/1_	EC-13-6/1_	EC-13-6/1_
40.87	39.48	40.85	40.98	40.62	40.71	40.80	39.52	40.37	40.74	40.83	40.90	40.32
0	0.004	0.014	0	0.028	0.058	0	0.003	0	0	0.004	0	0
0.19	0.51	0.40	0.45	0.58	0.61	0.45	0.34	0.43	0.45	0.48	0.42	0.51
0.03	0.01	0.02	0.00	0.01	0.00	0.02	0.03	0.01	0.02	0.00	0.00	0.01
0.08	0.06	0.11	0.09	0.12	0.14	0.10	0.09	0.09	0.08	0.10	0.06	0.08
15.17	11.63	10.62	11.88	9.68	10.42	12.29	15.35	11.32	10.10	10.04	11.74	10.19
43.26	49.76	48.17	44.69	49.96	48.64	46.31	45.39	47.54	48.38	48.80	45.05	49.09
0.00	0.00	0.00	0.00	0.00	0.00	0.02	0.01	0.00	0.00	0.00	0.00	0.01
0.25	0.16	0.16	0.16	0.18	0.18	0.17	0.27	0.16	0.14	0.18	0.16	0.12
0.00	0.00	0.00	0.00	0.01	0.00	0.02	0.00	0.00	0.00	0.02	0.00	0.00
0.01	0.00	0.00	0.08	0.00	0.22	0.19	0.00	0.00	0.00	0.00	0.22	0.20
99.86	101.60	100.33	98.33	101.18	100.97	100.36	101.01	99.91	99.92	100.45	98.54	100.54
83.6	88.4	89.0	87.0	90.2	89.3	87.0	84.1	88.2	89.5	89.7	87.2	89.6

Supplementary Table S5. (continued)

-		Type-3										Type-2
EC-13-6/1_	EC-13-6/1_	EC-13-6/1_	EC-13-6/1_	EC-13-6/1_	EC-13-6/1_	EC-13-6/1_	EC-13-6/1_	EC-13-6/1_	EC-13-6/1_	EC-13-6/1_	EC-13-6/1_	EC-13-6/1_
39.37	39.98	38.64	39.89	40.48	40.04	39.93	40.18	40.61	40.34	40.55	41.21	40.72
0	0.013	0	0	0.003	0	0	0.009	0	0	0	0	0
0.41	0.30	0.32	0.56	0.47	0.42	0.36	0.47	0.35	0.37	0.47	0.42	0.55
0.02	0.00	0.01	0.03	0.03	0.01	0.00	0.00	0.01	0.00	0.00	0.03	0.00
0.08	0.08	0.09	0.11	0.07	0.10	0.13	0.09	0.12	0.07	0.10	0.08	0.11
13.67	13.52	16.75	13.20	12.84	13.78	13.78	11.79	13.03	13.41	12.55	12.33	10.94
47.89	46.06	44.83	46.61	46.63	45.19	45.58	47.30	46.05	45.69	46.11	43.84	49.01
0.00	0.01	0.00	0.00	0.02	0.00	0.01	0.00	0.00	0.01	0.01	0.01	0.00
0.22	0.20	0.32	0.17	0.16	0.24	0.20	0.20	0.22	0.21	0.17	0.21	0.16
0.02	0.00	0.00	0.00	0.00	0.01	0.00	0.04	0.02	0.00	0.00	0.00	0.02
0.00	0.02	0.00	0.00	0.00	0.00	0.00	0.00	0.00	0.00	0.00	0.17	0.00
101.67	100.18	100.95	100.58	100.70	99.79	99.98	100.07	100.41	100.12	99.95	98.28	101.50
86.2	85.9	82.7	86.3	86.6	85.4	85.5	87.7	86.3	85.9	86.8	86.4	88.9

Supplementary Table S5. (continued)

				-		Type-1						
EC-13-6/1_	EC-13-6/1_	EC-13-6/1_	EC-13-6/1_	EC-13-6/1_	EC-13-6/1_	EC-13-6/1_	EC-13-6/1_	EC-13-6/1_	EC-13-6/1_	EC-13-6/1_	EC-13-6/1_	EC-13-6/1_
40.26	40.79	41.00	41.40	40.76	41.28	38.92	40.53	40.84	41.02	41.09	41.15	41.15
0.007	0	0.005	0.018	0.009	0	0.017	0	0	0	0.034	0	0.013
0.62	0.47	0.49	0.66	0.48	0.47	0.23	0.42	0.43	0.44	0.49	0.44	0.43
0.02	0.00	0.03	0.01	0.00	0.01	0.00	0.01	0.01	0.02	0.00	0.01	0.00
0.04	0.10	0.09	0.07	0.08	0.08	0.07	0.08	0.10	0.10	0.07	0.07	0.09
10.17	11.92	11.72	10.37	10.14	10.38	14.22	11.85	10.98	10.74	10.82	10.51	10.78
48.57	44.59	47.18	47.29	48.05	46.22	47.29	47.82	47.89	48.13	48.12	48.04	48.16
0.00	0.01	0.02	0.00	0.01	0.00	0.02	0.02	0.00	0.00	0.00	0.00	0.00
0.12	0.17	0.20	0.11	0.15	0.16	0.20	0.15	0.17	0.13	0.14	0.15	0.12
0.00	0.00	0.02	0.00	0.03	0.02	0.01	0.02	0.01	0.02	0.03	0.01	0.04
0.00	0.16	0.00	0.01	0.00	0.00	0.00	0.00	0.00	0.00	0.00	0.00	0.00
99.80	98.20	100.75	99.95	99.70	98.63	100.96	100.90	100.44	100.59	100.78	100.38	100.78
89.5	87.0	87.8	89.0	89.4	88.8	85.6	87.8	88.6	88.9	88.8	89.1	88.8

Supplementary Table S5. (continued)

			-					Type-1				
EC-13-6/1_	EC-13-6/1_	EC-13-6/1_	EC-13-6/1_	EC-13-6/1_	EC-13-6/1_	EC-13-6/1_	EC-13-6/1_	EC-13-6/1_	EC-13-6/1_	EC-13-6/1_	EC-13-6/1_	EC-13-6/1_
41.02	41.25	41.02	39.74	40.75	41.20	41.27	40.27	39.27	40.26	40.33	40.44	40.54
0	0	0	0	0	0.011	0	0	0.024	0	0.008	0	0.026
0.49	0.40	0.50	0.42	0.43	0.42	0.42	0.39	0.17	0.26	0.29	0.32	0.29
0.01	0.02	0.03	0.02	0.00	0.00	0.02	0.00	0.01	0.00	0.00	0.00	0.00
0.09	0.10	0.08	0.08	0.07	0.09	0.09	0.08	0.07	0.09	0.11	0.09	0.08
10.74	11.12	10.60	10.40	10.36	10.40	10.63	10.72	15.86	14.43	13.16	12.48	12.96
47.73	44.54	48.15	50.77	48.39	48.39	47.17	47.48	45.90	45.00	46.25	46.92	43.90
0.00	0.00	0.00	0.00	0.00	0.00	0.00	0.00	0.01	0.00	0.00	0.00	0.00
0.17	0.18	0.14	0.16	0.18	0.14	0.13	0.15	0.28	0.22	0.21	0.17	0.17
0.00	0.01	0.00	0.01	0.00	0.02	0.02	0.00	0.01	0.02	0.03	0.00	0.00
0.00	0.04	0.00	0.00	0.00	0.00	0.00	0.00	0.00	0.00	0.00	0.00	0.10
100.26	97.65	100.51	101.60	100.18	100.68	99.75	99.10	101.61	100.27	100.39	100.42	98.07
88.8	87.7	89.0	89.7	89.3	89.2	88.8	88.8	83.8	84.8	86.2	87.0	85.8

Supplementary Table S5. (continued)

	-						Type-1					
EC-13-6/1_	EC-13-6/1_	EC-13-6/1_	EC-13-6/1_	EC-13-6/1_	EC-13-6/1_	EC-13-6/1_	EC-13-6/1_	EC-13-6/1_	EC-13-6/1_	EC-13-6/1_	EC-13-6/1_	EC-13-6/1_
40.68	40.03	41.16	40.88	39.70	41.45	40.49	39.89	40.58	40.65	40.39	39.71	39.78
0.023	0.002	0	0	0	0.026	0	0	0.011	0	0.005	0.013	0
0.26	0.27	0.50	0.45	0.49	0.52	0.41	0.14	0.47	0.52	0.49	0.29	0.51
0.02	0.02	0.00	0.03	0.01	0.03	0.00	0.03	0.01	0.00	0.00	0.02	0.01
0.10	0.09	0.08	0.07	0.08	0.11	0.11	0.08	0.09	0.10	0.09	0.08	0.11
13.22	13.45	10.61	10.30	10.51	10.32	11.23	15.78	10.89	10.35	10.36	13.71	10.95
45.66	47.39	47.99	46.93	49.16	46.38	46.80	44.39	47.80	48.38	47.89	44.39	48.92
0.00	0.00	0.00	0.00	0.00	0.00	0.00	0.00	0.00	0.00	0.01	0.00	0.00
0.21	0.24	0.15	0.13	0.13	0.12	0.15	0.25	0.18	0.17	0.10	0.16	0.16
0.00	0.04	0.00	0.02	0.00	0.01	0.00	0.00	0.02	0.00	0.00	0.01	0.00
0.00	0.00	0.00	0.00	0.00	0.00	0.00	0.00	0.00	0.00	0.00	0.00	0.00
100.16	101.53	100.48	98.81	100.07	98.96	99.20	100.55	100.05	100.17	99.34	98.38	100.43
86.0	86.3	89.0	89.0	89.3	88.9	88.1	83.4	88.7	89.3	89.2	85.2	88.8

Supplementary Table S5. (continued)

	-					Type-1			-			
EC-13-6/1_	EC-13-6/1_	EC-13-6/1_	EC-13-6/1_	EC-13-6/1_	EC-13-6/1_	EC-13-6/1_	EC-13-6/1_	EC-13-6/1_	EC-13-6/1_	EC-13-6/1_	EC-13-6/1_	EC-13-6/1_
41.07	40.31	44.39	40.37	41.39	39.69	38.93	39.64	40.31	40.31	41.09	41.13	40.79
0.01	0.005	0	0.007	0	0	0	0	0.013	0	0	0	0.014
0.54	0.45	0.46	0.41	0.42	0.49	0.15	0.12	0.17	0.35	0.42	0.41	0.46
0.03	0.02	0.04	0.03	0.01	0.00	0.00	0.00	0.00	0.00	0.02	0.03	0.01
0.08	0.08	0.11	0.07	0.10	0.17	0.09	0.11	0.11	0.09	0.08	0.08	0.09
10.52	11.10	10.06	12.90	9.69	10.04	17.60	17.62	15.49	12.59	11.13	10.82	10.87
48.16	48.73	46.91	45.03	49.00	50.42	44.06	42.59	42.50	47.42	46.67	47.60	47.76
0.00	0.00	0.01	0.02	0.00	0.01	0.01	0.00	0.00	0.02	0.01	0.00	0.00
0.11	0.16	0.10	0.23	0.12	0.12	0.28	0.31	0.27	0.20	0.18	0.16	0.17
0.00	0.00	0.00	0.00	0.02	0.00	0.00	0.00	0.01	0.01	0.00	0.00	0.00
0.00	0.00	0.00	0.00	0.00	0.00	0.00	0.00	0.00	0.00	0.00	0.00	0.00
100.51	100.87	102.07	99.05	100.75	100.93	101.13	100.39	98.88	100.99	99.59	100.24	100.16
89.1	88.7	89.3	86.2	90.0	89.9	81.7	81.2	83.0	87.0	88.2	88.7	88.7

Supplementary Table S5. (continued)

	Type-2					-	Type-2					Type-1
EC-13-6/1_	EC-13-6/1_	EC-13-6/1_	EC-13-6/1_	EC-13-6/1_	EC-13-6/1_	EC-13-6/1_	EC-13-6/1_	EC-13-6/1_	EC-13-6/1_	EC-13-6/1_	EC-13-6/1_	EC-13-6/1_
40.91	40.34	40.64	40.97	41.02	40.00	39.58	39.89	40.57	40.13	40.73	40.19	40.67
0	0	0.019	0	0	0.037	0.036	0.016	0	0.005	0	0	0
0.44	0.38	0.34	0.39	0.44	0.35	0.31	0.53	0.45	0.43	0.49	0.64	0.48
0.03	0.02	0.01	0.02	0.00	0.02	0.05	0.00	0.01	0.02	0.02	0.00	0.00
0.07	0.09	0.07	0.11	0.11	0.12	0.08	0.10	0.11	0.08	0.09	0.08	0.10
10.83	11.10	10.27	10.30	10.60	9.95	12.03	11.74	9.94	10.02	10.00	10.70	11.53
45.61	48.22	48.90	47.95	46.03	48.86	49.46	48.08	48.79	49.14	48.45	46.43	47.56
0.00	0.00	0.00	0.00	0.02	0.00	0.00	0.00	0.00	0.00	0.00	0.00	0.00
0.16	0.15	0.18	0.12	0.16	0.17	0.16	0.18	0.14	0.12	0.14	0.15	0.14
0.02	0.00	0.01	0.00	0.00	0.00	0.01	0.00	0.00	0.00	0.02	0.03	0.02
0.09	0.00	0.00	0.00	0.00	0.00	0.00	0.00	0.00	0.00	0.00	0.00	0.00
98.15	100.30	100.45	99.86	98.38	99.51	101.72	100.53	100.01	99.93	99.93	98.21	100.49
88.2	88.6	89.5	89.2	88.6	89.7	88.0	87.9	89.7	89.7	89.6	88.6	88.0

Supplementary Table S5. (continued)

				Type-2					-	Type-2		
EC-13-6/1_	EC-13-6/1_	EC-13-6/1_	EC-13-6/1_	EC-13-6/1_	EC-13-6/1_	EC-13-6/1_	EC-13-6/1_	EC-13-6/1_	EC-13-6/1_	EC-13-6/1_	EC-13-6/1_	EC-13-6/1_
41.09	41.13	40.79	40.91	40.34	40.64	40.97	41.02	40.00	39.58	39.89	40.57	40.13
0	0	0.014	0	0	0.019	0	0	0.037	0.036	0.016	0	0.005
0.42	0.41	0.46	0.44	0.38	0.34	0.39	0.44	0.35	0.31	0.53	0.45	0.43
0.02	0.03	0.01	0.03	0.02	0.01	0.02	0.00	0.02	0.05	0.00	0.01	0.02
0.08	0.08	0.09	0.07	0.09	0.07	0.11	0.11	0.12	0.08	0.10	0.11	0.08
11.13	10.82	10.87	10.83	11.10	10.27	10.30	10.60	9.95	12.03	11.74	9.94	10.02
46.67	47.60	47.76	45.61	48.22	48.90	47.95	46.03	48.86	49.46	48.08	48.79	49.14
0.01	0.00	0.00	0.00	0.00	0.00	0.00	0.02	0.00	0.00	0.00	0.00	0.00
0.18	0.16	0.17	0.16	0.15	0.18	0.12	0.16	0.17	0.16	0.18	0.14	0.12
0.00	0.00	0.00	0.02	0.00	0.01	0.00	0.00	0.00	0.01	0.00	0.00	0.00
0.00	0.00	0.00	0.09	0.00	0.00	0.00	0.00	0.00	0.00	0.00	0.00	0.00
99.59	100.24	100.16	98.15	100.30	100.45	99.86	98.38	99.51	101.72	100.53	100.01	99.93
88.2	88.7	88.7	88.2	88.6	89.5	89.2	88.6	89.7	88.0	87.9	89.7	89.7

Supplementary Table S5. (continued)

		Type-1			Type-1			Type-1				-
EC-13-6/1_	EC-13-6/1_	EC-13-6/1_	EC-13-6/1_	EC-13-6/1_	EC-13-6/1_	EC-13-6/1_	EC-13-6/1_	EC-13-6/1_	EC-13-6/1_	EC-13-6/1_	EC-13-6/1_	EC-13-6/1_
40.73	40.19	40.67	40.20	38.55	40.48	40.95	38.81	38.93	40.14	40.25	40.16	40.79
0	0	0	0	0.004	0	0.025	0	0	0	0.013	0.027	0.003
0.49	0.64	0.48	0.30	0.09	0.30	0.36	0.15	0.14	0.29	0.34	0.26	0.52
0.02	0.00	0.00	0.01	0.02	0.02	0.00	0.03	0.03	0.03	0.00	0.00	0.00
0.09	0.08	0.10	0.08	0.14	0.11	0.08	0.11	0.10	0.09	0.08	0.09	0.10
10.00	10.70	11.53	12.93	18.32	14.33	10.66	15.88	17.03	12.92	12.67	13.11	11.07
48.45	46.43	47.56	45.71	41.13	44.89	47.73	43.95	43.43	46.47	46.46	44.93	47.48
0.00	0.00	0.00	0.00	0.00	0.00	0.01	0.01	0.00	0.00	0.00	0.00	0.00
0.14	0.15	0.14	0.19	0.37	0.23	0.19	0.28	0.34	0.20	0.20	0.17	0.15
0.02	0.03	0.02	0.00	0.00	0.02	0.03	0.00	0.00	0.03	0.01	0.00	0.02
0.00	0.00	0.00	0.00	0.00	0.00	0.00	0.00	0.00	0.00	0.00	0.00	0.00
99.93	98.21	100.49	99.41	98.63	100.37	100.03	99.22	100.00	100.17	100.01	98.75	100.13
89.6	88.6	88.0	86.3	80.0	84.8	88.9	83.1	82.0	86.5	86.7	85.9	88.4

Supplementary Table S5. (continued)

				Type-1						Type-2		
EC-13-6/1_	EC-13-6/1_	EC-13-6/1_	EC-13-6/1_	EC-13-6/1_	EC-13-6/1_	EC-13-6/1_	EC-13-6/1_	EC-13-6/1_	EC-13-6/1_	EC-13-6/1_	EC-13-6/1_	EC-13-6/1_
40.71	40.48	40.40	39.46	38.78	39.14	39.59	39.28	39.28	40.73	40.15	40.58	40.54
0	0.014	0.014	0	0	0	0	0.023	0.032	0.022	0.005	0	0
0.44	0.43	0.43	0.40	0.17	0.22	0.25	0.30	0.29	0.48	0.63	0.50	0.46
0.03	0.01	0.03	0.00	0.01	0.01	0.00	0.03	0.01	0.00	0.00	0.01	0.07
0.08	0.08	0.08	0.11	0.08	0.12	0.12	0.08	0.11	0.10	0.07	0.08	0.08
10.36	10.53	10.72	14.29	16.07	17.90	16.44	15.58	14.82	13.19	10.67	10.09	10.06
48.02	48.20	47.95	44.40	44.86	41.76	43.55	44.47	42.83	44.16	49.04	48.61	48.16
0.00	0.00	0.00	0.00	0.00	0.00	0.00	0.00	0.02	0.00	0.02	0.00	0.00
0.15	0.17	0.14	0.24	0.27	0.30	0.26	0.27	0.28	0.24	0.12	0.12	0.16
0.00	0.00	0.02	0.02	0.00	0.00	0.00	0.01	0.00	0.00	0.00	0.00	0.00
0.00	0.00	0.00	0.00	0.00	0.00	0.00	0.00	0.00	0.00	0.00	0.00	0.00
99.79	99.91	99.76	98.91	100.24	99.43	100.20	100.03	97.66	98.93	100.69	99.98	99.52
89.2	89.1	88.9	84.7	83.3	80.6	82.5	83.6	83.7	85.6	89.1	89.6	89.5

Supplementary Table S5. (continued)

					Type-2						
EC-13-6/1_	EC-13-6/1_	EC-13-6/1_	EC-13-6/1_	EC-13-6/1_	EC-13-6/1_	EC-13-6/1_	EC-13-6/1_	EC-13-6/1_	EC-13-6/1_	EC-13-6/1_	EC-13-6/1_
40.47	40.35	40.31	40.44	40.09	40.23	40.38	40.60	40.44	40.31	41.34	41.09
0	0.007	0	0.001	0	0	0	0	0	0.009	0.002	0
0.47	0.54	0.62	0.59	0.48	0.38	0.43	0.43	0.42	0.42	0.41	0.41
0.03	0.01	0.02	0.00	0.00	0.00	0.00	0.00	0.01	0.01	0.00	0.02
0.10	0.08	0.10	0.11	0.08	0.09	0.07	0.12	0.10	0.10	0.08	0.10
10.19	10.04	10.13	10.24	9.99	10.20	10.50	10.23	10.32	10.28	10.25	10.23
48.35	47.08	48.24	47.98	48.68	49.87	48.92	48.34	48.47	48.07	48.23	48.44
0.00	0.00	0.00	0.00	0.01	0.00	0.00	0.01	0.00	0.00	0.01	0.00
0.15	0.15	0.14	0.18	0.15	0.12	0.14	0.16	0.13	0.11	0.11	0.14
0.00	0.00	0.01	0.04	0.03	0.03	0.01	0.03	0.00	0.00	0.00	0.01
0.00	0.00	0.00	0.00	0.00	0.00	0.00	0.00	0.00	0.00	0.00	0.00
99.76	98.25	99.56	99.58	99.49	100.91	100.45	99.90	99.91	99.29	100.44	100.44
89.4	89.3	89.5	89.3	89.7	89.7	89.3	89.4	89.3	89.3	89.3	89.4

Supplementary Table S5. (continued)

Sample	EC-13-8/1										
Rock-type	B										
Analysis po	EC-13-8/1_	EC-13-8/1_	EC-13-8/1_	EC-13-8/1_	EC-13-8/1_	EC-13-8/1_	EC-13-8/1_	EC-13-8/1_	EC-13-8/1_	EC-13-8/1_	EC-13-8/1_
SiO_2 (wt.%	40.87	39.69	39.75	40.80	39.86	38.91	40.24	39.94	40.75	39.91	38.06
TiO_2	0	0	0	0.015	0	0.021	0	0	0	0	0
NiO	0.287	0.277	0.123	0.213	0.202	0.039	0.299	0.27	0.23	0.223	0.138
Al_2O_3	0.00	0.04	0.00	0.02	0.00	0.03	0.01	0.00	0.00	0.04	0.01
CaO	0.14	0.12	0.14	0.13	0.11	0.13	0.14	0.10	0.12	0.12	0.14
FeO*	10.75	11.70	14.53	11.34	12.06	22.40	10.72	11.15	11.15	11.97	18.08
MgO	46.05	47.36	43.73	44.37	45.94	36.10	46.80	46.86	45.72	46.24	40.92
K_2O	0.00	0.00	0.01	0.00	0.00	0.00	0.00	0.00	0.00	0.01	0.00
MnO	0.14	0.19	0.24	0.20	0.19	0.44	0.18	0.15	0.14	0.16	0.37
Na_2O	0.01	0.00	0.02	0.03	0.00	0.00	0.00	0.00	0.00	0.01	0.00
Cr_2O_3	0.0	0.0	0.0	0.0	0.0	0.0	0.0	0.0	0.0	0.0	0.0
P_2O_5	0.00	0.00	0.00	0.01	0.03	0.07	0.01	0.00	0.04	0.02	0.02
Total	98.28	99.39	98.57	97.15	98.40	98.14	98.42	98.48	98.15	98.71	97.74
Fo	88.4	87.8	84.3	87.5	87.2	74.2	88.6	88.2	88.0	87.3	80.1

Supplementary Table S5. (continued)

EC-13-8/1_	EC-13-8/1_	EC-13-8/1_	EC-13-8/1_	EC-13-8/1_	EC-13-8/1_	EC-13-8/1_	EC-13-8/1_	EC-13-8/1_	EC-13-8/1_	EC-13-8/1_	EC-13-8/1_
40.15	40.99	39.46	38.87	40.20	38.41	39.64	37.96	39.95	39.31	39.80	39.72
0	0	0	0	0	0.027	0	0.014	0.008	0	0.03	0
0.252	0.27	0.159	0.14	0.184	0.134	0.163	0.041	0.23	0.251	0.218	0.14
0.00	0.02	0.03	0.03	0.01	0.02	0.02	0.00	0.03	0.01	0.00	0.00
0.13	0.12	0.14	0.16	0.13	0.13	0.15	0.12	0.16	0.11	0.12	0.11
10.47	10.28	12.32	12.40	11.01	12.47	12.87	22.81	11.06	10.63	11.67	16.50
47.15	45.83	45.75	46.40	46.59	46.94	45.29	35.30	46.33	48.28	45.82	39.95
0.00	0.00	0.00	0.00	0.00	0.00	0.01	0.00	0.01	0.01	0.00	0.00
0.19	0.13	0.17	0.23	0.19	0.23	0.23	0.45	0.17	0.15	0.20	0.26
0.00	0.04	0.00	0.00	0.04	0.00	0.04	0.00	0.00	0.00	0.01	0.00
0.0	0.0	0.0	0.1	0.0	0.0	0.0	0.0	0.0	0.0	0.0	0.0
0.02	0.01	0.01	0.07	0.04	0.00	0.00	0.00	0.03	0.02	0.04	0.00
98.39	97.67	98.02	98.42	98.39	98.36	98.42	96.69	97.99	98.77	97.90	96.68
88.9	88.8	86.9	87.0	88.3	87.0	86.2	73.4	88.2	89.0	87.5	81.2

Supplementary Table S5. (continued)

EC-13-8/1_	EC-13-8/1_	EC-13-8/1_	EC-13-8/1_	EC-13-8/1_	EC-13-8/1_	EC-13-8/1_	EC-13-8/1_	EC-13-8/1_	EC-13-8/1_	EC-13-8/1_	EC-13-8/1_
40.15	40.99	39.46	38.87	40.20	38.41	39.64	37.96	39.95	39.31	39.80	39.72
0	0	0	0	0	0.027	0	0.014	0.008	0	0.03	0
0.252	0.27	0.159	0.14	0.184	0.134	0.163	0.041	0.23	0.251	0.218	0.14
0.00	0.02	0.03	0.03	0.01	0.02	0.02	0.00	0.03	0.01	0.00	0.00
0.13	0.12	0.14	0.16	0.13	0.13	0.15	0.12	0.16	0.11	0.12	0.11
10.47	10.28	12.32	12.40	11.01	12.47	12.87	22.81	11.06	10.63	11.67	16.50
47.15	45.83	45.75	46.40	46.59	46.94	45.29	35.30	46.33	48.28	45.82	39.95
0.00	0.00	0.00	0.00	0.00	0.00	0.01	0.00	0.01	0.01	0.00	0.00
0.19	0.13	0.17	0.23	0.19	0.23	0.23	0.45	0.17	0.15	0.20	0.26
0.00	0.04	0.00	0.00	0.04	0.00	0.04	0.00	0.00	0.00	0.01	0.00
0.0	0.0	0.0	0.1	0.0	0.0	0.0	0.0	0.0	0.0	0.0	0.0
0.02	0.01	0.01	0.07	0.04	0.00	0.00	0.00	0.03	0.02	0.04	0.00
98.39	97.67	98.02	98.42	98.39	98.36	98.42	96.69	97.99	98.77	97.90	96.68
88.9	88.8	86.9	87.0	88.3	87.0	86.2	73.4	88.2	89.0	87.5	81.2

Supplementary Table S5. (continued)

EC-13-8/1_	EC-13-8/1_	EC-13-8/1_	EC-13-8/1_	EC-13-8/1_	EC-13-8/1_	EC-13-8/1_	EC-13-8/1_	EC-13-8/1_	EC-13-8/1_	EC-13-8/1_	EC-13-8/1_
40.36	40.44	39.60	40.28	40.98	40.12	37.41	40.08	38.68	39.61	38.92	40.29
0.017	0.005	0.024	0	0.003	0.054	0	0	0.023	0.023	0.023	0
0.244	0.238	0.198	0.297	0.287	0.274	0.104	0.248	0.214	0.192	0.114	0.299
0.03	0.02	0.02	0.02	0.05	0.05	0.04	0.00	0.00	0.03	0.03	0.01
0.12	0.13	0.15	0.13	0.12	0.13	0.16	0.14	0.12	0.13	0.13	0.11
10.66	11.79	11.38	10.59	10.31	10.43	15.42	11.05	11.66	12.86	20.07	9.95
46.57	44.07	47.75	47.32	45.31	47.22	44.94	46.49	46.65	45.27	36.24	47.25
0.00	0.00	0.00	0.00	0.01	0.01	0.01	0.00	0.00	0.00	0.01	0.00
0.17	0.22	0.19	0.18	0.18	0.14	0.23	0.13	0.22	0.23	0.37	0.17
0.01	0.00	0.02	0.00	0.00	0.00	0.01	0.00	0.00	0.00	0.00	0.03
0.0	0.0	0.0	0.0	0.0	0.0	0.0	0.0	0.0	0.0	0.0	0.1
0.04	0.00	0.01	0.01	0.03	0.00	0.04	0.01	0.00	0.00	0.00	0.00
98.22	96.91	99.33	98.81	97.30	98.45	98.36	98.16	97.56	98.35	95.91	98.16
88.6	87.0	88.2	88.8	88.7	89.0	83.9	88.2	87.7	86.3	76.3	89.4

Supplementary Table S5. (continued)

EC-13-8/1_	EC-13-8/1_	EC-13-8/1_	EC-13-8/1_	EC-13-8/1_	EC-13-8/1_	EC-13-8/1_	EC-13-8/1_	EC-13-8/1_	EC-13-8/1_	EC-13-8/1_	EC-13-8/1_
39.93	36.10	40.01	36.62	39.89	39.07	39.23	36.86	40.32	39.93	40.05	39.90
0	0	0	0	0.005	0	0	0.002	0	0	0.001	0.019
0.273	0.121	0.252	0.038	0.244	0.228	0.143	0.155	0.257	0.266		0.262
0.03	0.33	0.03	0.02	0.03	0.00	0.00	0.00	0.00	0.07	0.00	0.03
0.12	0.16	0.11	0.18	0.15	0.13	0.13	0.15	0.10	0.12	0.12	0.10
10.79	13.72	10.57	23.73	10.73	10.78	13.02	14.27	10.50	10.14	10.48	10.38
46.71	45.83	46.66	34.84	46.30	45.94	44.86	45.58	46.23	46.53	47.47	47.36
0.01	0.00	0.00	0.00	0.00	0.01	0.00	0.00	0.01	0.01	0.01	0.01
0.15	0.21	0.17	0.49	0.16	0.15	0.27	0.27	0.18	0.19	0.13	0.14
0.00	0.04	0.00	0.00	0.01	0.00	0.01	0.03	0.00	0.00	0.00	0.02
0.0	0.0	0.0	0.0	0.0	0.0	0.0	0.0	0.0	0.1		0.0
0.01	0.07	0.00	0.06	0.01	0.00	0.00	0.01	0.02	0.01	0.04	0.07
98.03	96.58	97.83	95.98	97.53	96.32	97.67	97.32	97.66	97.39	98.29	98.28
88.5	85.6	88.7	72.3	88.5	88.4	86.0	85.1	88.7	89.1	89.0	89.1

Supplementary Table S5. (continued)

EC-13-8/1_	EC-13-8/1_	EC-13-8/1_	EC-13-8/1_	EC-13-8/1_	EC-13-8/1_	EC-13-8/1_	EC-13-8/1_	EC-13-8/1_	EC-13-8/1_	EC-13-8/1_	EC-13-8/1_
39.92	40.15	40.41	39.91	38.91	39.80	39.75	39.85	38.00	39.89	39.87	39.81
0.013	0	0.022	0	0.002	0.045	0	0.003	0	0.018	0	0.028
	0.301	0.263	0.266	0.052	0.272	0.289	0.275	0.02	0.242	0.205	0.215
0.00	0.01	0.02	0.04	0.02	0.03	0.01	0.03	0.02	0.03	0.02	0.00
0.11	0.11	0.20	0.11	0.18	0.13	0.11	0.12	0.13	0.15	0.14	0.13
10.39	10.29	10.35	10.51	21.99	10.45	10.65	10.42	22.68	11.01	11.55	11.58
47.09	47.41	46.63	46.60	35.19	47.11	47.45	47.04	34.52	46.47	46.02	46.15
0.00	0.00	0.01	0.00	0.00	0.00	0.00	0.00	0.01	0.01	0.00	0.02
0.17	0.18	0.20	0.19	0.46	0.20	0.16	0.13	0.50	0.18	0.17	0.19
0.02	0.00	0.03	0.00	0.00	0.01	0.00	0.00	0.00	0.01	0.00	0.00
	0.0	0.0	0.0	0.0	0.0	0.0	0.1	0.0	0.0	0.0	0.0
0.01	0.05	0.00	0.00	0.00	0.02	0.00	0.00	0.00	0.02	0.00	0.00
97.72	98.54	98.13	97.67	96.81	98.12	98.42	97.94	95.88	98.03	97.97	98.12
89.0	89.1	88.9	88.8	74.0	88.9	88.8	89.0	73.1	88.3	87.7	87.7

Supplementary Table S5. (continued)

EC-13-8/1_	EC-13-8/1_	EC-13-8/1_	EC-13-8/1_	EC-13-8/1_	EC-13-8/1_	EC-13-8/1_	EC-13-8/1_	EC-13-8/1_	EC-13-8/1_	EC-13-8/1_	EC-13-8/1_
39.79	39.60	40.27	40.23	39.97	40.48	39.94	39.71	39.33	40.21	40.13	39.99
0	0.005	0	0.005	0	0	0	0	0.029	0.027	0	0.025
0.24	0.164	0.31	0.262	0.299	0.227	0.295	0.2	0.129	0.338	0.345	0.367
0.03	0.00	0.03	0.04	0.01	0.04	0.01	0.01	0.00	0.04	0.01	0.03
0.12	0.16	0.10	0.13	0.11	0.12	0.13	0.11	0.12	0.11	0.14	0.12
10.80	12.15	9.84	10.69	10.31	10.20	9.97	11.11	14.31	9.88	9.40	9.27
46.69	45.79	47.79	46.76	46.86	47.19	47.21	46.13	43.50	47.15	47.51	47.65
0.01	0.00	0.00	0.00	0.01	0.00	0.00	0.00	0.01	0.00	0.00	0.01
0.16	0.19	0.12	0.16	0.16	0.16	0.12	0.16	0.20	0.14	0.15	0.15
0.00	0.01	0.02	0.02	0.00	0.02	0.03	0.01	0.00	0.02	0.00	0.03
0.0	0.0	0.0	0.0	0.0	0.0	0.0	0.0	0.0	0.0	0.0	0.0
0.00	0.03	0.01	0.00	0.04	0.02	0.00	0.03	0.02	0.03	0.00	0.03
97.83	98.09	98.52	98.30	97.76	98.47	97.72	97.47	97.63	97.95	97.70	97.65
88.5	87.0	89.6	88.6	89.0	89.2	89.4	88.1	84.4	89.5	90.0	90.2

Supplementary Table S5. (continued)

EC-13-8/1_	EC-13-8/1_	EC-13-8/1_	EC-13-8/1_	EC-13-3/4_	EC-13-3/4_	EC-13-3/4_	EC-13-3/4_	EC-13-3/4_	EC-13-3/4_	EC-13-3/4_	EC-13-3/4_
39.58	39.92	40.16	40.13	38.15	38.42	35.81	37.99	38.27	38.25	39.43	38.50
0.025	0	0	0	0	0.01	0	0.031	0	0	0	0.007
0.299	0.253	0.268	0.245	0.118	0.147	0.152	0.19	0.172	0.151	0.138	0.11
0.00	0.00	0.04	0.03	0.06	0.03	0.01	0.03	0.00	0.01	0.00	0.01
0.12	0.10	0.13	0.11	0.15	0.14	0.09	0.11	0.13	0.14	0.17	0.10
9.48	10.66	10.49	10.61	20.09	18.70	18.87	18.78	18.68	19.11	19.98	20.39
48.77	46.43	46.81	46.60	38.60	39.93	40.60	40.36	40.16	39.69	38.74	38.23
0.00	0.00	0.00	0.00	0.00	0.01	0.00	0.00	0.00	0.00	0.00	0.01
0.16	0.17	0.17	0.17	0.35	0.32	0.33	0.35	0.33	0.30	0.34	0.39
0.00	0.00	0.02	0.03	0.00	0.00	0.00	0.01	0.03	0.00	0.00	0.04
0.0	0.0	0.1	0.0	0.0	0.0	0.0	0.0	0.0	0.0	0.0	0.5
0.03	0.00	0.00	0.00	0.00	0.00	0.00	0.01	0.00	0.00	0.00	0.00
98.47	97.57	98.17	97.94	97.51	97.70	95.87	97.84	97.78	97.70	98.81	98.24
90.2	88.6	88.8	88.7	77.4	79.2	79.3	79.3	79.3	78.7	77.6	77.0

Supplementary Table S5. (continued)

EC-13-3/4_	EC-13-3/4_	EC-13-3/4_	EC-13-8/1_	EC-13-8/1_	EC-13-8/1_	EC-13-8/1_	EC-13-8/1_	EC-13-8/1_	EC-13-8/1_	EC-13-8/1_	EC-13-8/1_
38.02	38.09	37.63	39.59	40.94	41.18	40.93	39.86	40.37	40.43	39.30	40.67
0.009	0	0.041	0.02	0.00	0.00	0.00	0.00	0.00	0.02	0.01	0.00
0.136	0.121	0.072	0.092	0.211	0.331	0.278	0.283	0.246	0.163	0.132	0.129
0.04	0.00	0.02	0.00	0.03	0.01	0.04	0.00	0.02	0.00	0.00	0.03
0.16	0.11	0.17	0.12	0.13	0.13	0.09	0.13	0.13	0.09	0.13	0.17
18.97	18.76	22.70	15.83	11.39	10.16	10.57	11.83	11.95	13.00	18.96	15.41
40.31	39.82	36.91	44.48	47.73	46.67	47.08	47.38	46.17	45.36	39.88	42.55
0.01	0.00	0.00	0.00	0.00	0.00	0.00	0.01	0.00	0.00	0.00	0.00
0.32	0.33	0.39	0.26	0.19	0.17	0.14	0.17	0.18	0.22	0.27	0.27
0.01	0.00	0.02	0.01	0.00	0.00	0.00	0.03	0.00	0.00	0.00	0.00
0.0	0.0	0.0	0.0	0.0	0.0	0.0	0.0	0.0	0.0	0.0	0.0
0.02	0.00	0.02	-	-	-	-	-	-	-	-	-
98.01	97.24	97.98	100.41	100.63	98.65	99.11	99.69	99.05	99.28	98.68	99.23
79.1	79.1	74.3	83.4	88.2	89.1	88.8	87.7	87.3	86.1	78.9	83.1

Supplementary Table S5. (continued)

Sample	EC-13-8/1										
Rock-type	B										
Analysis po	EC-13-8/1_	EC-13-8/1_	EC-13-8/1_	EC-13-8/1_	EC-13-8/1_	EC-13-8/1_	EC-13-8/1_	EC-13-8/1_	EC-13-8/1_	EC-13-8/1_	EC-13-8/1_
SiO_2 (wt.%	40.87	39.69	39.75	40.80	39.86	38.91	40.24	39.94	40.75	39.91	38.06
TiO_2	0	0	0	0.015	0	0.021	0	0	0	0	0
NiO	0.287	0.277	0.123	0.213	0.202	0.039	0.299	0.27	0.23	0.223	0.138
Al_2O_3	0.00	0.04	0.00	0.02	0.00	0.03	0.01	0.00	0.00	0.04	0.01
CaO	0.14	0.12	0.14	0.13	0.11	0.13	0.14	0.10	0.12	0.12	0.14
FeO*	10.75	11.70	14.53	11.34	12.06	22.40	10.72	11.15	11.15	11.97	18.08
MgO	46.05	47.36	43.73	44.37	45.94	36.10	46.80	46.86	45.72	46.24	40.92
K_2O	0.00	0.00	0.01	0.00	0.00	0.00	0.00	0.00	0.00	0.01	0.00
MnO	0.14	0.19	0.24	0.20	0.19	0.44	0.18	0.15	0.14	0.16	0.37
Na_2O	0.01	0.00	0.02	0.03	0.00	0.00	0.00	0.00	0.00	0.01	0.00
Cr_2O_3	0.0	0.0	0.0	0.0	0.0	0.0	0.0	0.0	0.0	0.0	0.0
P_2O_5	0.00	0.00	0.00	0.01	0.03	0.07	0.01	0.00	0.04	0.02	0.02
Total	98.28	99.39	98.57	97.15	98.40	98.14	98.42	98.48	98.15	98.71	97.74
Fo	88.4	87.8	84.3	87.5	87.2	74.2	88.6	88.2	88.0	87.3	80.1

Supplementary Table S5. (continued)

Sample	EC-13-3/4										
Rock-type	HMB										
Analysis po	EC-13-3/4_	EC-13-3/4_	EC-13-3/4_	EC-13-3/4_	EC-13-3/4_	EC-13-3/4_	EC-13-3/4_	EC-13-3/4_	EC-13-3/4_	EC-13-3/4_	EC-13-3/4_
SiO_2 (wt.%	38.18	37.88	37.34	39.13	38.69	38.56	37.67	39.49	39.49	38.06	38.21
TiO_2	0	0.005	0.023	0	0	0.021	0	0.006	0	0	0
NiO	0.124	0.114	0.099	0.137	0.21	0.201	0.121	0.206	0.232	0.181	0.176
Al_2O_3	0.01	0.00	0.01	0.02	0.00	0.00	0.00	0.00	0.01	0.02	0.02
CaO	0.15	0.18	0.17	0.14	0.13	0.17	0.16	0.14	0.13	0.13	0.13
FeO*	20.65	22.06	23.48	15.26	18.59	18.87	21.55	12.53	12.17	18.88	20.12
MgO	42.28	41.24	39.64	46.59	42.77	43.16	40.94	48.99	49.26	43.27	42.35
K_2O	0.00	0.00	0.00	0.00	0.00	0.00	0.01	0.01	0.00	0.00	0.01
MnO	0.37	0.42	0.44	0.24	0.27	0.35	0.41	0.18	0.18	0.30	0.35
Na_2O	0.01	0.01	0.02	0.00	0.05	0.00	0.04	0.01	0.00	0.02	0.01
Cr_2O_3	0.01	0.00	0.00	0.00	0.00	0.00	0.01	0.00	0.00	0.00	0.00
P_2O_5	0.06	0.03	0.02	0.00	0.03	0.00	0.00	0.00	0.01	0.01	0.01
Total	101.82	101.95	101.23	101.50	100.75	101.32	100.90	101.56	101.48	100.87	101.38
Fo	78.5	76.9	75.1	84.5	80.4	80.3	77.2	87.5	87.8	80.3	79.0

Supplementary Table S5. (continued)

EC-13-3/4_	EC-13-3/4_	EC-13-3/4_	EC-13-3/4_	EC-13-3/4_	EC-13-3/4_	EC-13-3/4_	EC-13-3/4_	EC-13-3/4_	EC-13-3/4_	EC-13-3/4_	EC-13-3/4_
37.40	39.07	38.35	38.98	39.06	39.70	38.80	38.72	38.96	39.33	37.84	39.50
0.021	0	0.008	0	0.02	0.014	0	0.013	0	0	0.016	0
0.145	0.165	0.124	0.122	0.205	0.18	0.145	0.107	0.206	0.209	0.132	0.207
0.18	0.03	0.00	0.00	0.01	0.01	0.04	0.01	0.00	0.00	0.01	0.00
0.13	0.12	0.15	0.13	0.15	0.14	0.18	0.17	0.16	0.14	0.14	0.15
20.98	15.77	20.56	15.48	13.09	17.42	18.97	20.11	16.51	14.99	20.87	14.87
41.19	46.43	41.99	46.39	48.26	43.83	43.53	41.03	45.35	46.97	41.41	47.03
0.00	0.02	0.01	0.00	0.01	0.00	0.01	0.00	0.01	0.00	0.00	0.01
0.41	0.27	0.41	0.25	0.21	0.30	0.34	0.33	0.26	0.28	0.40	0.25
0.03	0.01	0.01	0.01	0.03	0.00	0.00	0.00	0.00	0.00	0.03	0.01
0.00	0.00	0.00	0.00	0.01	0.00	0.00	0.00	0.05	0.00	0.01	0.01
0.05	0.04	0.01	0.04	0.00	0.00	0.00	0.01	0.02	0.00	0.02	0.00
100.53	101.93	101.62	101.40	101.05	101.59	102.01	100.50	101.51	101.92	100.88	102.03
77.8	84.0	78.4	84.2	86.8	81.8	80.4	78.4	83.0	84.8	78.0	84.9

Supplementary Table S5. (continued)

EC-13-3/4_	EC-13-3/4_	EC-13-3/4_	EC-13-3/4_	EC-13-3/4_	EC-13-3/4_	EC-13-3/4_	EC-13-3/4_	EC-13-3/4_	EC-13-3/4_	EC-13-3/4_	EC-13-3/4_
38.69	38.39	38.41	38.56	39.50	38.85	38.13	38.40	38.56	38.66	39.70	38.68
0.015	0	0	0.028	0	0.055	0.006	0	0.014	0.028	0.003	0
0.111	0.13	0.173	0.135	0.136	0.18	0.069	0.091	0.12	0.157	0.253	0.131
0.00	0.01	0.00	0.00	0.02	0.01	0.00	0.00	0.00	0.00	0.04	0.00
0.13	0.15	0.15	0.12	0.15	0.13	0.14	0.16	0.17	0.14	0.12	0.12
21.08	21.74	18.55	17.95	19.83	18.10	22.17	21.47	18.69	17.76	12.13	17.72
41.43	41.04	43.58	44.21	40.57	44.16	40.80	41.28	43.65	44.52	48.89	43.86
0.00	0.00	0.00	0.00	0.00	0.00	0.01	0.00	0.00	0.00	0.00	0.00
0.39	0.34	0.34	0.32	0.35	0.35	0.39	0.34	0.31	0.28	0.16	0.28
0.00	0.02	0.00	0.00	0.00	0.00	0.03	0.00	0.01	0.01	0.00	0.00
0.00	0.00	0.00	0.03	0.00	0.00	0.00	0.00	0.00	0.00	0.00	0.00
0.02	0.05	0.02	0.00	0.04	0.01	0.00	0.04	0.02	0.01	0.03	0.00
101.87	101.87	101.21	101.35	100.59	101.86	101.74	101.77	101.55	101.55	101.32	100.79
77.8	77.1	80.7	81.4	78.5	81.3	76.6	77.4	80.6	81.7	87.8	81.5

Supplementary Table S5. (continued)

EC-13-3/4_	EC-13-3/4_	EC-13-3/4_	EC-13-3/4_	EC-13-3/4_	EC-13-3/4_!
38.82	38.31	38.38	38.92	38.95	37.54
0	0	0	0.022	0	0
0.111	0.157	0.098	0.209	0.227	0.198
0.04	0.00	0.01	0.00	0.07	0.03
0.13	0.13	0.14	0.12	0.12	0.13
16.96	20.24	21.57	16.67	16.17	20.20
44.85	41.98	40.98	44.69	43.43	43.44
0.01	0.00	0.00	0.02	0.01	0.01
0.25	0.41	0.35	0.26	0.25	0.36
0.00	0.05	0.00	0.00	0.00	0.00
0.02	0.01	0.00	0.01	0.14	0.00
0.06	0.03	0.00	0.00	0.02	0.02
101.24	101.32	101.52	100.92	99.39	101.93
82.5	78.7	77.2	82.7	82.7	79.3

Sample	EC-13-1/1			
Rock-type	HAB			
Analysis po	EC-13-1/1_	EC-13-1/1_	EC-13-1/1_	EC-13-1_1-
SiO_2 (wt.%	38.85	39.14	39.98	38.58
TiO_2	0	0	0.047	0
NiO	0.119	0.074	0.053	0.124
Al_2O_3	0.03	0.04	0.00	0.02
CaO	0.27	0.22	0.13	0.13
FeO*	17.83	18.18	19.81	18.01
MgO	44.02	43.91	40.05	45.53
K_2O	0.017	0.025	0.031	0.000
MnO	0.26	0.29	0.30	0.30
Na_2O	0.04	0.00	0.01	0.05
Cr_2O_3	0.00	0.00	0.19	0.04
P_2O_5	0.00	0.00	0.00	0.01
Total	101.45	101.89	100.61	102.80
Fo	81.5	81.2	78.3	81.8

Supplementary Table S5. (continued)

EC-13-1_1-	EC-13-1_1-	EC-13-1_1-	EC-13-1_1-	EC-13-1_1-	EC-13-1_1-	EC-13-1_1-	EC-13-1_1-	EC-13-1_1-	EC-13-1_1-	EC-13-1_1-	EC-13-1_1-
39.37	38.58	39.47	39.62	39.54	35.38	39.26	39.93	39.84	39.85	40.39	40.80
0.012	0	0	0.006	0.006	0.471	0	0.008	0.013	0	0.026	0
0.139	0.096	0.075	0.101	0.17	0.067	0.082	0.086	0.079	0.136	0.107	0.091
0.05	0.01	0.00	0.00	0.04	1.76	0.01	0.04	0.03	0.04	0.00	0.03
0.12	0.12	0.14	0.13	0.11	0.10	0.10	0.11	0.14	0.17	0.17	0.13
16.94	20.65	19.74	17.31	17.39	17.78	18.91	18.14	18.40	16.86	17.67	19.68
44.85	41.51	43.30	45.18	45.22	39.09	44.11	43.07	43.42	46.08	44.29	40.66
0.000	0.005	0.014	0.000	0.000	0.001	0.004	0.006	0.000	0.000	0.004	0.000
0.23	0.43	0.38	0.25	0.26	0.25	0.27	0.29	0.31	0.27	0.28	0.43
0.00	0.02	0.01	0.02	0.00	0.00	0.02	0.00	0.04	0.00	0.03	0.00
0.00	0.00	0.00	0.10	0.06	2.58	0.04	0.01	0.09	0.18	0.20	0.00
0.02	0.00	0.00	0.02	0.02	0.00	0.00	0.01	0.01	0.00	0.00	0.00
101.72	101.41	103.15	102.77	102.81	97.56	102.81	101.69	102.41	103.58	103.16	101.80
82.5	78.2	79.6	82.3	82.3	79.7	80.6	80.9	80.8	83.0	81.7	78.6

Supplementary Table S5. (continued)

EC-13-1_1-	EC-13-1_1-	EC-13-1_1-	EC-13-1_1-	EC-13-1_1-	EC-13-1_1-	EC-13-1_1-	EC-13-1_1-	EC-13-1_1-	EC-13-1_1-	EC-13-1_1-	EC-13-1_1-
40.37	39.91	40.35	40.64	40.20	40.37	40.55	40.95	41.24	40.43	40.75	40.69
0.003	0.008	0	0	0	0.001	0.011	0.011	0.025	0	0.002	0.033
0.129	0.136	0.17	0.081	0.073	0.079	0.066	0.16	0.104	0.158	0.051	0.08
0.02	0.00	0.00	0.00	0.04	0.00	0.02	0.04	0.00	0.00	0.02	0.00
0.09	0.12	0.13	0.10	0.12	0.12	0.13	0.11	0.17	0.14	0.16	0.17
16.76	16.73	16.25	17.91	19.12	19.82	19.80	16.30	17.49	17.20	17.44	18.90
45.59	45.46	45.87	43.70	43.53	41.15	41.42	45.82	43.66	45.62	44.65	43.52
0.000	0.001	0.003	0.005	0.000	0.009	0.001	0.000	0.000	0.018	0.000	0.001
0.23	0.22	0.24	0.29	0.37	0.38	0.32	0.20	0.29	0.26	0.25	0.34
0.00	0.00	0.00	0.01	0.00	0.01	0.00	0.02	0.03	0.00	0.00	0.00
0.00	0.00	0.01	0.02	0.04	0.03	0.03	0.00	0.01	0.02	0.03	0.09
0.00	0.00	0.00	0.00	0.00	0.01	0.02	0.04	0.00	0.06	0.00	0.01
103.18	102.58	103.02	102.75	103.50	101.98	102.37	103.66	103.02	103.90	103.35	103.82
82.9	82.9	83.4	81.3	80.2	78.7	78.9	83.4	81.7	82.5	82.0	80.4

Supplementary Table S5. (continued)

EC-13-1_1-	EC-13-1_1-	EC-13-1_1-	EC-13-1_1-	EC-13-1_1-	EC-13-1_1-	EC-13-1_1-	EC-13-1_1-	EC-13-1_1-	EC-13-1_1-	EC-13-1_1-	EC-13-1_1-
40.37	39.91	40.35	40.64	40.20	40.37	40.55	40.95	41.24	40.43	40.75	40.69
0.003	0.008	0	0	0	0.001	0.011	0.011	0.025	0	0.002	0.033
0.129	0.136	0.17	0.081	0.073	0.079	0.066	0.16	0.104	0.158	0.051	0.08
0.02	0.00	0.00	0.00	0.04	0.00	0.02	0.04	0.00	0.00	0.02	0.00
0.09	0.12	0.13	0.10	0.12	0.12	0.13	0.11	0.17	0.14	0.16	0.17
16.76	16.73	16.25	17.91	19.12	19.82	19.80	16.30	17.49	17.20	17.44	18.90
45.59	45.46	45.87	43.70	43.53	41.15	41.42	45.82	43.66	45.62	44.65	43.52
0.000	0.001	0.003	0.005	0.000	0.009	0.001	0.000	0.000	0.018	0.000	0.001
0.23	0.22	0.24	0.29	0.37	0.38	0.32	0.20	0.29	0.26	0.25	0.34
0.00	0.00	0.00	0.01	0.00	0.01	0.00	0.02	0.03	0.00	0.00	0.00
0.00	0.00	0.01	0.02	0.04	0.03	0.03	0.00	0.01	0.02	0.03	0.09
0.00	0.00	0.00	0.00	0.00	0.01	0.02	0.04	0.00	0.06	0.00	0.01
103.18	102.58	103.02	102.75	103.50	101.98	102.37	103.66	103.02	103.90	103.35	103.82
82.9	82.9	83.4	81.3	80.2	78.7	78.9	83.4	81.7	82.5	82.0	80.4

Supplementary Table S5. (continued)

EC-13-1_1-	EC-13-1_1-	EC-13-1_1-	EC-13-1_1-	EC-13-1_1-	EC-13-1_1-	EC-13-1_1-	EC-13-1_1-	EC-13-1_1-	EC-13-1_1-	EC-13-1_1-	EC-13-1_1-
40.37	39.91	40.35	40.64	40.20	40.37	40.55	40.95	41.24	40.43	40.75	40.69
0.003	0.008	0	0	0	0.001	0.011	0.011	0.025	0	0.002	0.033
0.129	0.136	0.17	0.081	0.073	0.079	0.066	0.16	0.104	0.158	0.051	0.08
0.02	0.00	0.00	0.00	0.04	0.00	0.02	0.04	0.00	0.00	0.02	0.00
0.09	0.12	0.13	0.10	0.12	0.12	0.13	0.11	0.17	0.14	0.16	0.17
16.76	16.73	16.25	17.91	19.12	19.82	19.80	16.30	17.49	17.20	17.44	18.90
45.59	45.46	45.87	43.70	43.53	41.15	41.42	45.82	43.66	45.62	44.65	43.52
0.000	0.001	0.003	0.005	0.000	0.009	0.001	0.000	0.000	0.018	0.000	0.001
0.23	0.22	0.24	0.29	0.37	0.38	0.32	0.20	0.29	0.26	0.25	0.34
0.00	0.00	0.00	0.01	0.00	0.01	0.00	0.02	0.03	0.00	0.00	0.00
0.00	0.00	0.01	0.02	0.04	0.03	0.03	0.00	0.01	0.02	0.03	0.09
0.00	0.00	0.00	0.00	0.00	0.01	0.02	0.04	0.00	0.06	0.00	0.01
103.18	102.58	103.02	102.75	103.50	101.98	102.37	103.66	103.02	103.90	103.35	103.82
82.9	82.9	83.4	81.3	80.2	78.7	78.9	83.4	81.7	82.5	82.0	80.4

Supplementary Table S5. (continued)

EC-13-1_1-	EC-13-1_1-	EC-13-1_1-	EC-13-1_1-	EC-13-1_1-	EC-13-1_1-	EC-13-1_1-	EC-13-1_1-	EC-13-1_1-	EC-13-1_1-	EC-13-1_1-	EC-13-1_1-
40.85	40.17	40.60	39.92	40.94	39.72	40.40	39.57	37.41	40.85	40.68	39.36
0	0.008	0	0.006	0	0.013	0	0.027	0.018	0.016	0	0
0.093	0.084	0.114	0.146	0.108	0.069	0.114	0.06	0.087	0.169	0.169	0.049
0.02	0.00	0.00	0.00	0.04	0.00	0.01	0.00	0.01	0.02	0.02	0.00
0.13	0.12	0.12	0.14	0.11	0.11	0.15	0.13	0.11	0.14	0.12	0.13
16.84	18.75	16.82	16.60	17.22	23.38	18.16	20.28	13.65	15.94	17.00	21.87
45.20	44.08	45.27	46.76	45.05	39.95	38.26	42.89	41.79	45.96	45.12	41.14
0.000	0.000	0.000	0.000	0.000	0.000	0.016	0.011	0.000	0.000	0.000	0.003
0.24	0.34	0.28	0.25	0.29	0.58	0.33	0.43	0.21	0.26	0.29	0.40
0.02	0.00	0.00	0.03	0.02	0.00	0.00	0.00	0.03	0.00	0.04	0.00
0.12	0.00	0.01	0.02	0.00	0.02	0.01	0.05	0.00	0.02	0.00	0.00
0.02	0.02	0.07	0.06	0.00	0.00	0.03	0.00	0.04	0.03	0.01	0.01
103.53	103.57	103.27	103.93	103.78	103.85	97.48	103.44	93.34	103.39	103.44	102.96
82.7	80.7	82.7	83.4	82.3	75.3	79.0	79.0	84.5	83.7	82.6	77.0

Supplementary Table S5. (continued)

EC-13-1_1-	EC-13-1_1-	EC-13-1_1-	EC-13-1_1-	EC-13-1_1-	EC-13-1_1-	EC-13-1_1-	EC-13-1_1-	EC-13-1_1-	EC-13-1_1-	EC-13-1_1-	EC-13-1_1-
41.19	40.74	41.49	40.98	41.22	37.27	40.38	41.18	40.40	41.20	41.21	40.86
0	0.024	0	0.002	0.005	0.067	0.012	0.017	0.029	0	0.029	0.047
0.086	0.114	0.12	0.056	0.088	0.103	0.122	0.115	0.099	0.147	0.132	0.132
0.01	0.00	0.04	0.00	0.11	0.55	0.03	0.01	0.04	0.00	0.02	0.01
0.13	0.10	0.12	0.13	0.13	0.21	0.11	0.12	0.13	0.15	0.13	0.12
18.97	16.19	17.09	19.72	16.33	16.38	17.15	16.95	19.35	16.47	16.15	16.86
42.98	45.85	43.97	42.81	44.03	47.11	45.31	45.14	43.54	45.79	45.75	45.49
0.005	0.000	0.005	0.005	0.008	0.019	0.000	0.001	0.000	0.000	0.000	0.000
0.34	0.23	0.27	0.37	0.23	0.23	0.29	0.25	0.38	0.27	0.23	0.24
0.03	0.03	0.00	0.00	0.00	0.02	0.00	0.01	0.00	0.00	0.00	0.02
0.00	0.00	0.00	0.05	0.18	0.53	0.00	0.00	0.00	0.00	0.00	0.02
0.00	0.02	0.04	0.03	0.03	0.00	0.01	0.04	0.00	0.03	0.00	0.00
103.75	103.30	103.14	104.16	102.36	102.49	103.40	103.83	103.97	104.05	103.65	103.79
80.2	83.5	82.1	79.5	82.8	83.7	82.5	82.6	80.0	83.2	83.5	82.8

Supplementary Table S5. (continued)

EC-13-1_1-	EC-13-1_1-	EC-13-1_1-	EC-13-1_1-
41.08	41.04	40.23	40.93
0.011	0.026	0	0.035
0.083	0.208	0.065	0.172
0.01	0.01	0.01	0.01
0.13	0.12	0.18	0.13
17.89	15.78	21.05	17.37
44.26	45.67	41.65	44.86
0.000	0.000	0.000	0.003
0.30	0.22	0.47	0.37
0.00	0.01	0.00	0.03
0.00	0.00	0.00	0.00
0.04	0.04	0.00	0.04
103.82	103.13	103.66	103.94
81.5	83.8	77.9	82.2

Additional References

81. Foley, S.F. *et al*. Minor and trace elements in olivines as probes into early igneous and mantle melting processes. *Earth Planet. Sci. Lett.* **363**, 181–191 (2013).

82. Takahashi, E. Origin of basaltic magmas: Implication from peridotite melting experiments and an olivine fractionation model. *Bull. Volcanol. Soc. Japan.* **30**, 17–40 (1986).
